# Oleanolic acid stimulation of cell migration involves a biphasic signaling mechanism

**DOI:** 10.1038/s41598-022-17553-w

**Published:** 2022-09-05

**Authors:** Javier Stelling-Férez, José Antonio Gabaldón, Francisco José Nicolás

**Affiliations:** 1 Department of Nutrition and Food Technology, Health Sciences PhD Program, Universidad Católica de Murcia (UCAM), Campus de los Jerónimos nº135, Guadalupe, 30107 Murcia, Spain; 2 Regeneration, Molecular Oncology and TGF-ß, Instituto Murciano de Investigación Biosanitaria (IMIB)-Arrixaca, Hospital Clínico Universitario Virgen de la Arrixaca, El Palmar, Murcia, Spain

**Keywords:** Biochemistry, Cell biology, Drug discovery, Molecular biology

## Abstract

Cell migration is a critical process for wound healing, a physiological phenomenon needed for proper skin restoration after injury. Wound healing can be compromised under pathological conditions. Natural bioactive terpenoids have shown promising therapeutic properties in wound healing. Oleanolic acid (OA), a triterpenoid, enhances in vitro and in vivo cell migration. However, the underlying signaling mechanisms and pathways triggered by OA are poorly understood. We have previously shown that OA activates epidermal growth factor receptor (EGFR) and downstream effectors such as mitogen-activated protein (MAP) kinase cascade and c-Jun N-terminal kinase (JNK), leading to c-Jun transcription factor phosphorylation, all of which are involved in migration. We performed protein expression or migration front protein subcellular localization assays, which showed that OA induces c-Jun activation and its nuclear translocation, which precisely overlaps at wound-edge cells. Furthermore, c-Jun phosphorylation was independent of EGFR activation. Additionally, OA promoted actin cytoskeleton and focal adhesion (FA) dynamization. In fact, OA induced the recruitment of regulator proteins to FAs to dynamize these structures during migration. Moreover, OA changed paxillin distribution and activated focal adhesion kinase (FAK) at focal adhesions (FAs). The molecular implications of these observations are discussed.

## Introduction

Wound healing is a physiological phenomenon that involves different cell types, which mainly carry out proliferation, differentiation and migration. These processes are tightly regulated for proper wound closure and skin barrier restoration. Taking this into account, wound healing is a process that is often compromised under pathological conditions associated with aging and illness, thus resulting in an incomplete wound closure, and frequently turning into a chronic wound^1^. Therefore, the search for active natural compounds capable of enhancing cell migration, and consequently wound healing, must be considered an alternative interesting option to solve this problem. Oleanolic acid (OA), a natural triterpenoid found in a large number of plants^2^, is known for its beneficial properties in wound healing: it accelerates wound healing with better aesthetic results^3^, it increases the tensile strength of wounds^4^, and it also has ulcer healing properties^4–7^. Recently, several authors have reported a negative effect of OA on cell migration^8–11^, see “Discussion” for further details. Nevertheless, we and others have shown that OA is able to promote cell migration in Mv1Lu and MDA-MB-231 cell lines^12^ and in mouse fibroblasts^6^. However, the molecular mechanisms behind this process are not yet fully understood.

Cell migration is important during wound closure to reshape the skin epithelium and restore its integrity^13,14^. Cell migration is the cells’ response to a variety of cytokines and other molecular messengers through proteins responsible for integrating these signals: mitogen-activated protein (MAP) kinases^14^. Recently, Bernabé et al.^12^ showed that OA triggers different cell-migration-related molecular mechanisms by using Mv1Lu and MDA-MB-231 cells. Thus, in both mentioned cell lines, OA induces the activation of extracellular signal-regulated kinases (ERK1/2) and c-Jun N-terminal kinases (JNK1/2), with c-Jun overexpression and activation by phosphorylation as a consequence^12^. JNK^15,16^ and MAP kinase /ERK kinase, kinase 1^17,18^, have been shown to be essential for cell migration. Indeed, MEK1 (ERK1/2 kinase) and JNK1/2 specific inhibitors prevent OA-stimulated cell migration in both Mv1Lu and MDA-MB-231 cells^12^. On the other hand, OA also stimulates Epidermal growth factor receptor (EGFR) phosphorylation in a protein-synthesis independent fashion^12^ in MDA-MB-231 cells. Actually, in this later cell line, specific inhibition of EGFR by PD153035 produces a severe halt of OA-stimulated cell migration and so does an EGFR blocking antibody^12^. Moreover, OA treatment causes the cell internalization of EGFR similarly to its ligand, EGF^12^.

Cell migration^13,14^ is a very well-coordinated process that requires a directional organized movement of cells^19^. Besides the rapid change of actin filaments^20^, the formation and disassembly of cell adhesion sites are required^21,22^. Therefore, it is observed in actively migrating cells at the leading edge of epithelia^19^. Cytoskeleton and focal adhesions (FAs) are integrated into a dynamic machinery in continuous change, with many actin-cytoskeleton interacting proteins involved^20,22,23^. One of them is paxillin, a cytosolic adaptor protein that coordinates the process^24^ and binds to other proteins that constitute FAs^25^. FAs are macromolecular complexes which interact with the extracellular matrix (ECM) through integrins^26^. FAs integrate multiple regulation pathways, detecting mechanical signals that allow cells to migrate^27^. In these multiprotein complexes, paxillin is essential to integrate all these signals and is found bound to regulatory kinases that are necessary to manage actin dynamics. Paxillin Ser 178 is phosphorylated by c-Jun N-terminal kinase (JNK) and this residue is critical for paxillin regulation of cell migration^28^.

Focal adhesion kinase (FAK) is another important paxillin regulator. FAK is a non-receptor tyrosine kinase combining both integrin and growth-factor signals on cell migration^29^, and it plays a key role in cell migration dynamics^22,30–33^. FAK operates as the cell master controller for FAs remodeling at the migration leading edge, consequently driving directional cell movement^22,34^. There is evidence that FAK stimulation occurs when growth factors bind to tyrosine kinase receptors, especially EGFR^35^. Indeed, activation of EGFR has been linked to phosphorylation of different residues at FAK with the phosphorylation of Tyr 925 as the final consequence^35^. FAK Tyr 925 phosphorylation is critical for its activating function, and it is induced by cell integrin assembling and growth factor stimulation^31,36^. FAK also modulates focal adhesion assembly and disassembly by the phosphorylation of different proteins, including paxillin^22,33,37^.

In this paper, we narrowed down the molecular and subcellular events that happen in the cell in the presence of OA. By using differential time read-out and specific inhibitors, we came across a complex and interesting molecular mechanism, involving EGFR and possibly receptors lacking intrinsic tyrosine-kinase activity. We also studied the effect of OA on cytoskeleton reorganization and focal adhesion dynamics at the wound-edge of the healing scratch.

## Results

### Oleanolic acid over-expresses c-Jun and promotes its activation at the wound edge during migration

**Figure 1:**
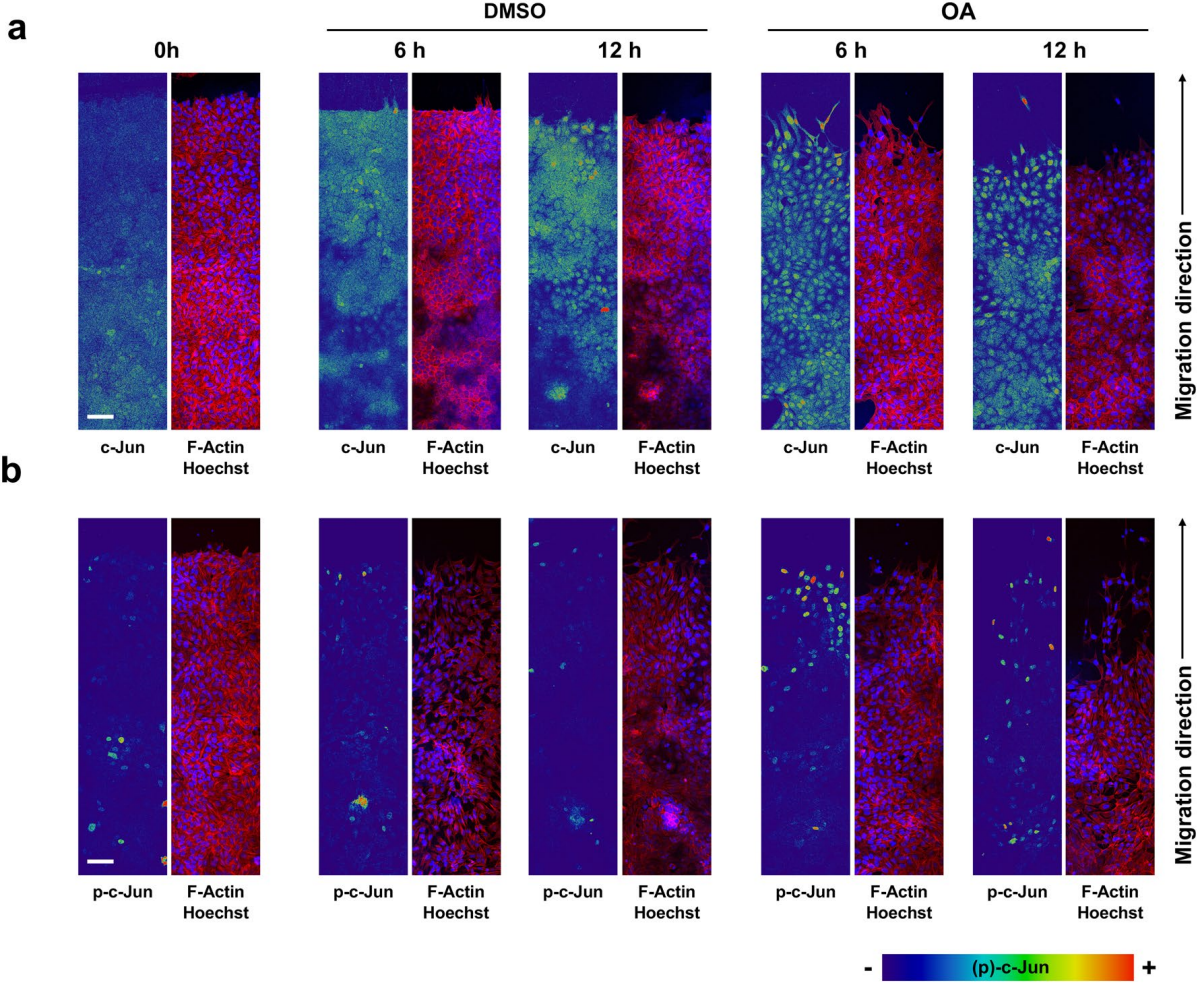
Oleanolic acid induces the expression of c-Jun and promotes its phosphorylation at the edge of wound-scratched Mv1Lu cells. Confluent Mv1Lu cells were scratched and allowed to migrate for the indicated times. Cells were treated with 5 µM OA or DMSO equivalent volume (control). Cells were immunostained with specific antibodies against c-Jun (**a**) and its active phosphorylated form, p-c-Jun (**b**). Co-staining with phalloidin and Hoechst-33258 was used to show actin cytoskeleton and nuclei, respectively. Images of c-Jun/p-c-Jun fluorescence were converted into pseudo-color with ImageJ software to show the intensity of c-Jun staining. Color rainbow scale represents fluorescence intensity for either c-Jun or phospho-c-Jun. Actin fibers (F-actin): red. Nuclei: blue. Images were obtained with a confocal microscope. This experiment was repeated at least three times. Representative images are shown. Scale bar indicates 50 µm.

C-Jun is overexpressed in response to OA stimulation^12^ in non-malignant mink lung epithelial cells, Mv1Lu. This cell line is recognized as a good epithelial model for the study of cell motility due to its ability to migrate and also to stop proliferation when the cells reach confluence^38–40^. Mv1Lu cells were stimulated with OA during a wound healing scratch assay. The evolution of the migration front was monitored and the expression of c-Jun was studied by immunostaining. The advantage of this approach is to directly study proteins involved in OA enhanced migration, focusing on its topographic and subcellular localization along the wound edge. The presence of OA produced a neat overexpression of c-Jun at the wound edge, which was significantly high after 6 h (Fig. 1a; Supplemental Fig. 1a).

Also, upon OA treatment (6 h), levels of phospho-c-Jun, regarded as phospho-Ser 63, were significantly high at the wound edge (Fig. 1b; Supplemental Fig. 1b). Remarkably, c-Jun and p-c-Jun overexpression occurred at the nucleus of actively migrating cells. Although c-Jun and p-c-Jun levels were enhanced at the migration edge of Mv1Lu, there was a clear decrease in the c-Jun protein expression and its active form as cells were further away from the wound edge. The visualization of actin, by using phalloidin staining (Supplemental Fig. 2), revealed critical changes in the intensity of the cytoskeleton, which were very evident after OA stimulation.

All these data suggest that the effect of OA is especially powerful at wound-edge cells, activating and overexpressing c-Jun protein there, which correlates well with the improvement of cell migration exhibited by OA^12^.

### Activation of different OA targets exhibits different timing

**Figure 2:**
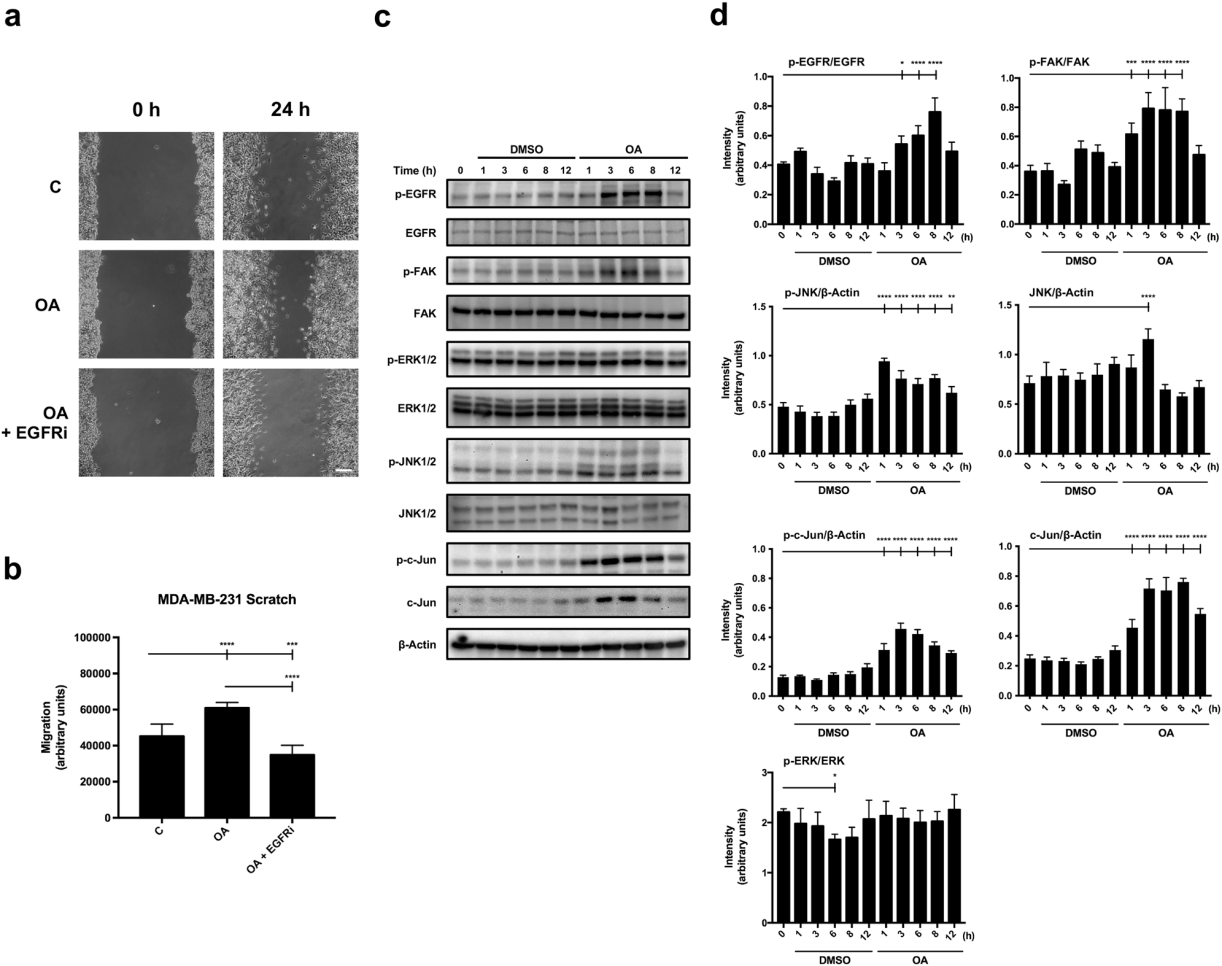
C-Jun and EGFR OA activation, necessary for OA-cell migration, are decoupled in MDA-MB-231 cells. (**a**) The figure shows representative images of cell migration obtained under control conditions compared to those with 10 µM OA and OA plus 2,5 µM EGFRi after 24 h treatment. Scale bar 200 µm. (**b**) Plot represents cell migration as the difference obtained between the quantified areas at time 0 h and time 24 h in each condition. Asterisks indicate statistically significant differences between the selected conditions according to a one-way ANOVA statistical analysis: (*p < 0.05, **p < 0.005, ***p < 0.001 and ****p < 0.0001). (**c**) Total protein extracts from serum-starved sub-confluent MDA-MB-231 cells treated with 10 µM OA, EGF or DMSO equivalent volume as vehicle control. Different proteins were assayed at the indicated times (h) targeting phospho-FAK (Tyr 925), phospho-EGFR (Tyr 1068), phospho-ERK1/2 (Thr 202/Tyr 204), phospho-JNK1/2 (Thr 183/Tyr 185), phospho-c-Jun (Ser 63). Additionally, total protein expression was assayed: EGFR, FAK, ERK1/2, JNK1/2 and c-Jun. β-Actin was used as a loading control. A representative experiment is shown. (**d**) Plots with intensity values of each protein assayed by Western-blot, by gathering the data of three independent experiments. Intensity values were quantified and collected by ImageJ software. Asterisks indicate statistically significant differences between the selected conditions according to a one-way ANOVA statistical analysis: (*p < 0.05, **p < 0.005, ***p < 0.001 and ****p < 0.0001).

Previously, it has been shown that OA produces an activation of EGFR in MDA-MB-231 cells that is independent of protein translation^12^. MDA-MB-231 cells are a good model for cell migration^41–43^ and exhibit a good expression of EGFR^44^. Indeed, EGFR inhibitor (EGFRi), PD153035, was capable of completely reducing OA-stimulated migration of MDA-MB-231 (Fig. 2a,b)^12^. It is important to measure the effect of OA at the protein signaling level. By using sub-confluent cells, we can interpret what the effect of OA might be on the wound edge^12,45^. To gain further knowledge of the activation of EGFR by OA and its involvement in the activation of previously described protein targets^12^, we conducted an OA-induction and monitored it at different times. Immediately after stimulation with OA (1 h), the activation by phosphorylation of JNK1/2 and c-Jun was detected together with the total level increase of both proteins (Fig. 2c,d). Strikingly, when looking at EGFR, we could detect its OA activation at three hours, but no sign of activation was seen at earlier time points. So, compared with c-Jun or JNK1/2, activation of EGFR showed a delayed stimulation by OA (Fig. 2c,d). Moreover, c-Jun phosphorylation experienced a further increase at three hours, which may suggest a double response and different phosphorylation dynamics for both proteins, c-Jun and EGFR. Coherently with this, the activation of JNK kinase seemed to be also biphasic, showing an early activation and a later (6 h) higher activation.

Activation of EGFR has been linked to phosphorylation of different residues at the Focal Adhesion Kinase (FAK), which leads to the Tyr 925 phosphorylation^35^. Tyr 925 phosphorylation of FAK is a key event on the management of cell migration^46^. Interestingly, OA produced the phosphorylation of FAK with a time dynamic parallel to the phosphorylation of EGFR (Fig. 2c,d).

Altogether, these results suggest that OA stimulates different targets to activate several key elements related to cell migration showing a clear time-uncoupling for different proteins.

### Uncoupled timing activation of c-Jun and EGFR can also be seen in Mv1Lu cells

**Figure 3:**
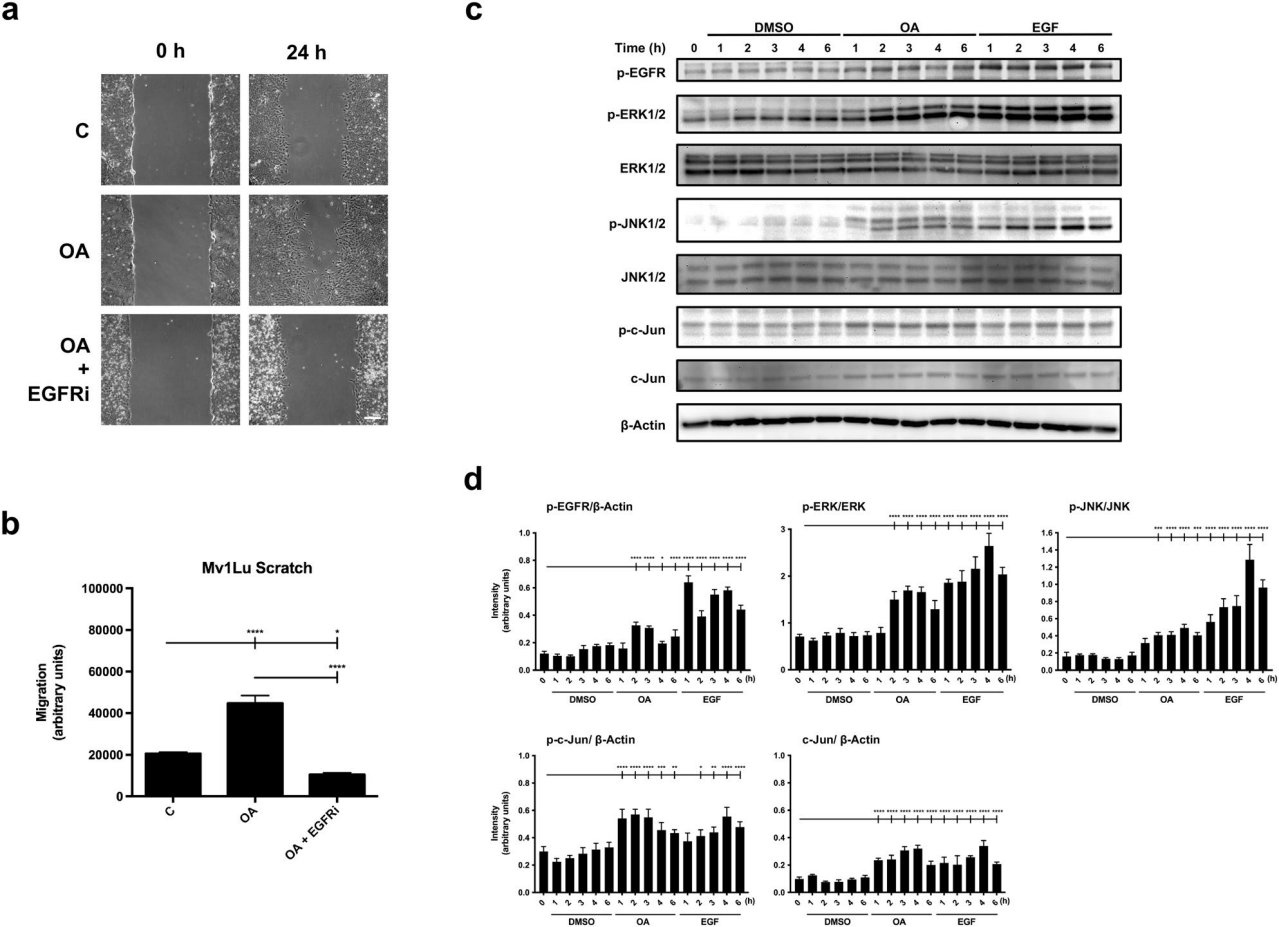
C-Jun and EGFR OA activation, necessary for OA-cell migration, are decoupled in Mv1Lu cells. (**a**) The figure shows representative images of cell migration obtained under control conditions compared to those with 5 µM OA and OA plus 2,5 µM EGFRi after 24 h treatment. Scale bar 200 µm. (**b**) Plot represents cell migration as the difference obtained between the quantified areas at time 0 h and time 24 h in each condition. Asterisks indicate statistically significant differences between the selected conditions according to a one-way ANOVA statistical analysis: (*p < 0.05, **p < 0.005, ***p < 0.001 and ****p < 0.0001). (**c**) Total protein extracts from serum-starved sub-confluent Mv1Lu cells treated with 5 µM OA, EGF or DMSO equivalent volume as vehicle control. Different protein phosphorylation was assayed at the indicated times (h) targeting phospho-EGFR (Tyr 1068), phospho-ERK1/2 (Thr 202/Tyr 204), phospho-JNK1/2 (Thr 183/Tyr 185) and phospho-c-Jun (Ser 63). Additionally, total protein expression was assayed: ERK1/2, JNK1/2 and c-Jun. β-Actin was used as a loading control. A representative experiment is shown. (**d**) Plots with intensity values of each protein assayed by Western-blot, by gathering the data of three independent experiments. Asterisks indicate statistically significant differences between the selected conditions according to a one-way ANOVA statistical analysis: (*p < 0.05, **p < 0.005, ***p < 0.001 and ****p < 0.0001).

Mv1Lu cells were also stimulated to migrate by OA^12^. Moreover, Mv1Lu OA stimulated migration was severely inhibited by EGFRi (Fig. 3a,b)^12^. A time course experiment was conducted to investigate the sequence of events. In this case, and similarly to MDA-MB-231 cells, we observed an early (1 h) c-Jun phosphorylation and total protein level increase compared with a later activation of EGFR (2 h) (Fig. 3c,d). Likewise, JNK1/2 phosphorylation could be detected 1 h after OA activation, being enhanced from 2 h onwards. In a similar trend, we could observe that ERK was phosphorylated by OA not earlier than 2 h following the same dynamics than EGFR phosphorylation, and therefore suggesting that ERK OA activation could be dependent on EGFR OA activation.

These data support the evidence that, upon OA treatment, c-Jun is activated before EGFR, thus suggesting a primary induction triggered by OA on this transcription factor through an alternative pathway different from the one activating EGFR. Also, these data, together with MDA-MB-231 data, support the possibility that, at least, two independent OA stimulated pathways converge on the activation of c-Jun and therefore are involved in OA-stimulated cell migration.

### The use of specific inhibitors against EGFR, MEK and JNK confirms an EGFR independent OA-induced pathway in MDA-MB-231 cells

**Figure 4:**
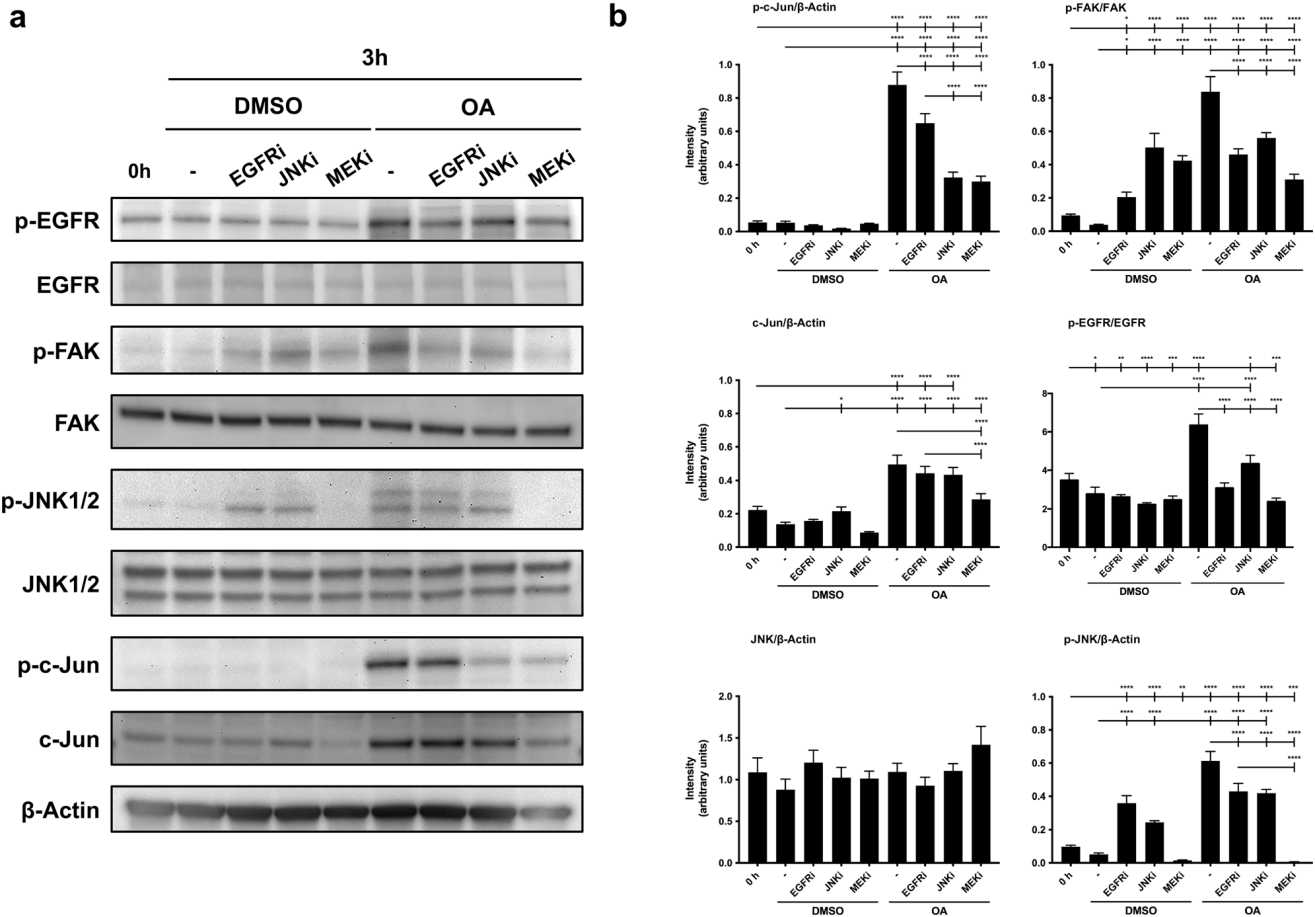
The use of specific inhibitors against EGFR, MEK and JNK upon OA treatment reveals a new OA-induced pathway in MDA-MB-231 cells. (**a**) Total protein extracts from serum-starved sub-confluent MDA-MB-231 cells, treated with 10 µM OA, EGF or DMSO. Besides, cells were treated 30 min before with the following inhibitors in the absence or presence of OA: 2.5 µM EGFRi, 50 µM MEKi and 15 µM JNKi. Different proteins’ phosphorylation was assayed at the indicated times (h): phospho-FAK (Tyr 925), phospho-EGFR (Tyr 1068), phospho-ERK1/2 (Thr 202/Tyr 204), phospho-JNK1/2 (Thr 183/Tyr 185) and phospho-c-Jun (Ser 63). Additionally, total protein expression was assayed: EGFR, FAK, JNK1/2 and c-Jun. β-Actin was used as a loading control. A representative experiment is shown. (**b**) Plots with intensity values of each protein assayed by Western-blot, by gathering the data of three independent experiments. Intensity values were quantified and collected by ImageJ software. Asterisks indicate statistically significant differences between the selected conditions according to a one-way ANOVA statistical analysis: (*p < 0.05, **p < 0.005, ***p < 0.001 and ****p < 0.0001).

The study of OA-activation of different proteins showed time uncoupling between c-Jun phosphorylation and EGFR activation. To assess whether there was an effective independence of both phenomena, cells were stimulated with OA in the presence of a specific inhibitor for EGFR, PD153035 (EGFRi). Although EGFRi importantly reduced OA-stimulated phosphorylation of EGFR, a minute effect was perceived on c-Jun phosphorylation or JNK1/2 phosphorylation. However, the presence of JNK1/2 inhibitor SP600125 (JNKi) caused a drastic negative effect on c-Jun phosphorylation (Fig. 4a,b). When the phosphorylation of FAK was measured in these conditions, the clear activation by OA was inhibited by the presence of EGFRi, drastically reducing the phosphorylation of FAK. The use of PD98059, a MAPK inhibitor (MEKi), produced a clear abrogation of both c-Jun and FAK phosphorylation (Fig. 4a,b). All these data suggest a complex and intricate signaling network that is interconnected to activate several proteins in response to OA. Nevertheless, a clear independence of c-Jun phosphorylation could be seen in the activation of EGFR phosphorylation upon OA stimulation, while FAK activation was dependent on EGFR activity.

**Figure 5:**
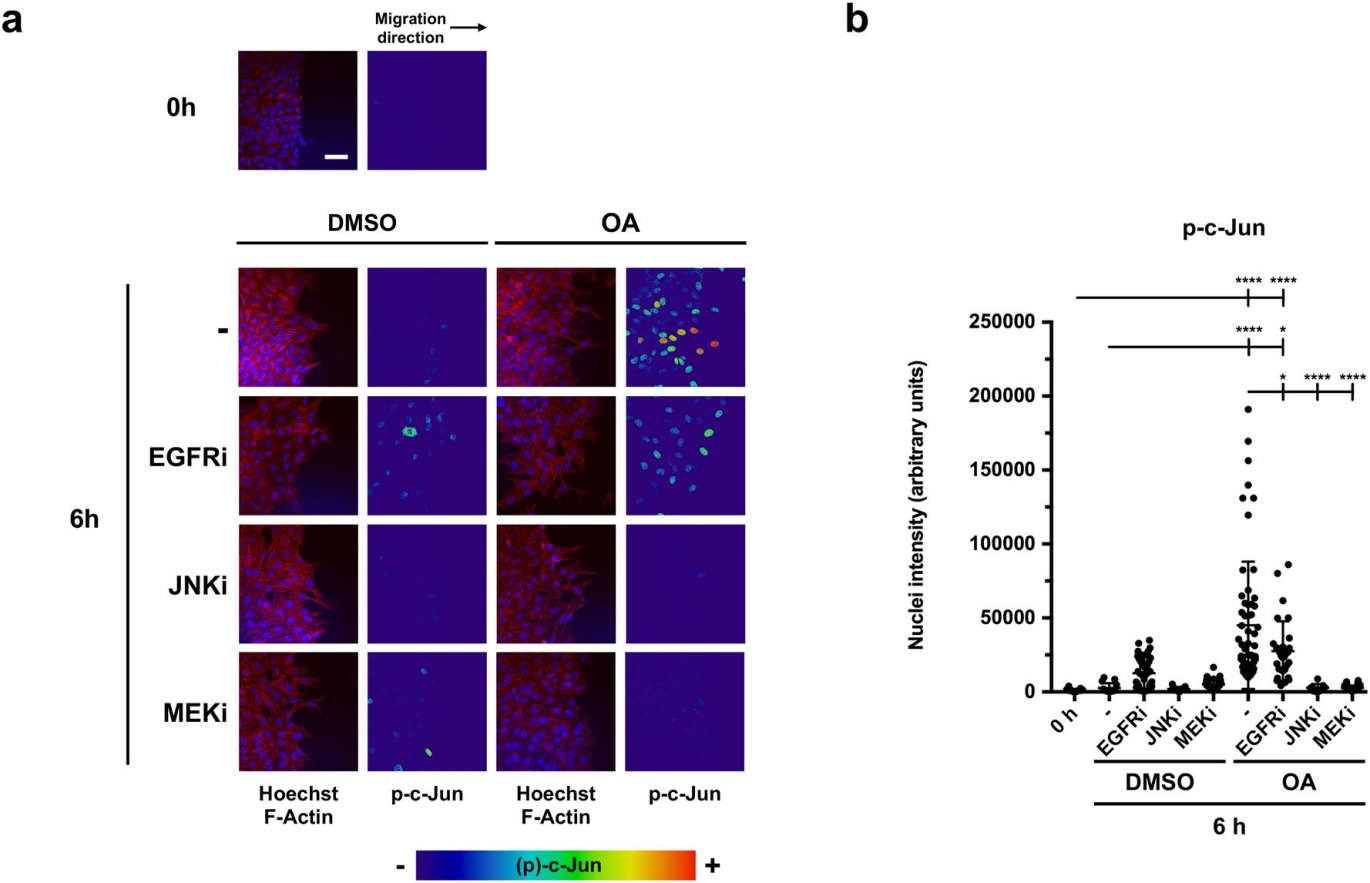
Specific inhibitors against EGFR, MEK and JNK, upon OA treatment, show the independent OA-activation on c-Jun at the edge of wound-scratched Mv1Lu cells at wound edge. (**a**) Confluent Mv1Lu cells were scratched and allowed to migrate for 6 h. Cells treated with 5 µM OA or DMSO equivalent volume were immunostained with specific antibodies against c-Jun transcription factor. Additionally, cells were treated 30 min before with the following inhibitors: 2.5 µM EGFRi, 50 µM MEKi and 15 µM JNKi. Co-staining with phalloidin and Hoechst-33258 was used to show actin cytoskeleton and nuclei, respectively. Images of p-c-Jun fluorescence were converted into pseudo-color with ImageJ software to show the intensity of c-Jun staining. Color rainbow scale represents fluorescence intensity for phospho-c-Jun. Actin fibers (F-actin): red. Nuclei: blue. Images were obtained with a confocal microscope. This experiment was repeated at least three times. Representative images are shown. Scale bar indicates 10 µm. (**b**) Plot represents the data obtained by p-c-Jun intensity at cell nuclei. In every condition, each point on the plot represents p-c-Jun intensity at the nucleus of one cell, quantified by ImageJ software. With the collected data of p-c-Jun intensity, a one-way ANOVA statistical analysis was performed (*p < 0.05, **p < 0.005, ***p < 0.001 and ****p < 0.0001).

We wanted to assess the effect of the different inhibitors previously used on the activation of the same proteins upon OA stimulation at Mv1Lu cells. In this scenario, the stimulation with OA produced a late activation of ERK that was inhibited with EGFRi or MEKi but not with JNKi. The activation of c-Jun, however, was not prevented by EGFRi (Supplemental Fig. 3a,b). The activation (phosphorylation) of c-Jun after OA stimulation overlapped with migrating cells at the wound-healing scratch assay front (Fig. 5a,b). Strikingly, both JNKi and MEKi prevented the phosphorylation of c-Jun. On the other hand, the presence of EGFRi enabled the activation of c-Jun further supporting the fact that c-Jun activation by OA was not dependent on EGFR activation. All these data reinforced the notion that the activation of c-Jun by OA in different cell lines has, at least, two distinct non-related mechanisms, one of which does not require EGFR activation.

### OA promotes changes in actin fibers and paxillin distribution

In cell migration, actin protein is essential for building a suitable dynamic architecture that allows cells to migrate^23,47,48^. As shown before, actin varies in response to OA in the migrating cells (see Supplemental Fig. 2). Actin interacts, among others, with paxillin, which is an adaptor protein, playing a role in cytoskeleton anchoring to focal adhesion (FA) and its remodeling during cell migration^24,27,37,49^. One of the consequences of OA stimulation was the phosphorylation of paxillin at Ser 178 (Supplemental Fig. 4a,b). JNK regulates cell migration through the phosphorylation of paxillin at Ser 178^50^, and indeed JNK is activated in response to OA (see Fig. 2c,d). Both phosphorylation of JNK and phosphorylation of paxillin were coincidental in time, thus suggesting a correlation between both phenomena and the activation by OA.

**Figure 6:**
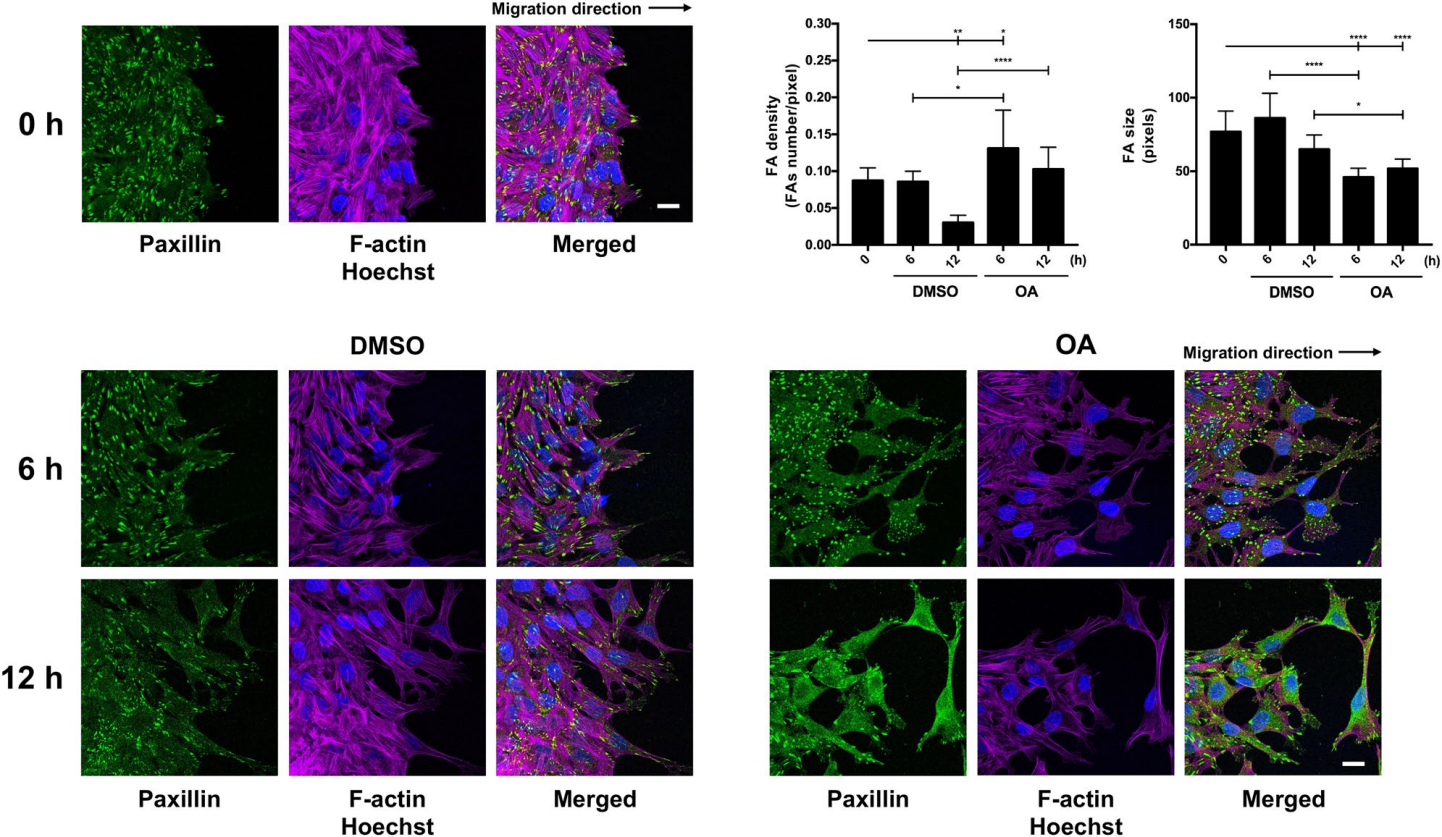
OA promotes changes in FAs and actin cytoskeleton. Confluent Mv1Lu cells were scratched and allowed to migrate for 6 and 12 h. Cells treated with 5 µM OA or DMSO equivalent volume (vehicle control) were immunostained with specific antibodies against paxillin (green). Co-staining with phalloidin and Hoechst-33258 was used to show actin cytoskeleton (magenta) and nuclei (blue), respectively. This experiment was repeated at least three times. Representative images are shown. Scale bar indicates 10 µm. Quantification of the density of FA as FA number per filopodia area. Quantification of FA size (average size) at the filopodia area. One-way ANOVA statistical analysis was performed (*p < 0.05, **p < 0.005, ***p < 0.001 and ****p < 0.0001).

To further understand the effect of OA on cell migration, we immunostained paxillin in wound-healing scratched Mv1Lu cells. When immediately-scratched cells were observed, paxillin was distributed as compacted focal adhesions (Fig. 6). As soon as 6 h after stimulation with OA, paxillin staining pattern changed to display smaller FA and denser distribution. This was especially noticeable at places where the cells were forming lamellipodia or filopodia (Fig. 6; Supplemental Fig. 5). Again, as seen before (see Supplemental Fig. 1), changes in actin intensity were very evident 6 h after OA stimulation (Fig. 6; Supplemental Fig. 5), became more apparent after 12 h after OA treatment. This correlated well with the fact that OA enhances cell migration^12^. All these observations suggest that OA produces high dynamization of cytoskeleton and FA compatible with a higher migration rate.

In a previous publication, we showed that EGFRi, JNKi or MEKi had an inhibitory effect on OA-stimulated cell migration^12^. To further comprehend the consequences of using these inhibitors, we studied the subcellular actin architecture of the cells upon OA stimulation in the presence of these inhibitors. Both OA and EGF produced clear changes in cell architecture losing actin stress fibers, which were still evident with JNKi and MEKi but not with EGFRi (Supplemental Fig. 6a). Concerning FAs, the use of MEKi and EGFRi decreased their density and increased their size, although the effect of EGFRi was milder size-wise (Supplemental Fig. 6b,c). In contrast, in response to OA, neither the size nor the density of FA seemed to be affected by the presence of JNKi.

### OA treatment produces FAK activation and promotes focal adhesion remodeling in scratched Mv1Lu cells

**Figure 7:**
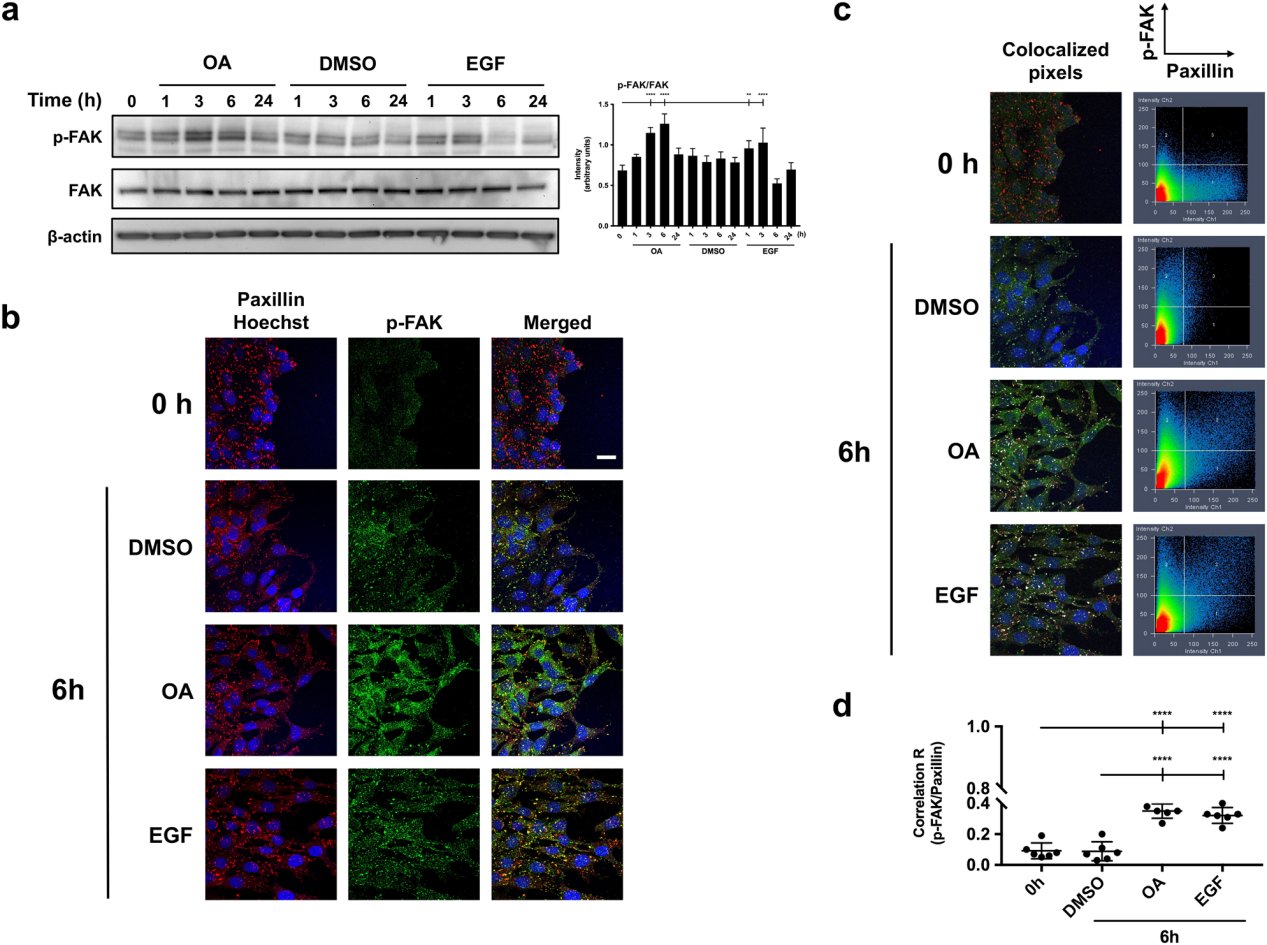
OA induces FAK phosphorylation and promotes its localization in focal adhesion with paxillin in Mv1Lu cells. (**a**) The activation of FAK was assessed by Western Blot at Tyr 925, at the indicated times in the presence of 5 µM OA, DMSO equivalent volume or EGF. Additionally, total protein expression was assayed: FAK. β-Actin was used as a loading control. Phospho-FAK intensity values in Western-blot were quantified by ImageJ to conduct a one-way ANOVA analysis showed on the plot (*p < 0.05, **p < 0.005, ***p < 0.001 and ****p < 0.0001). (**b**) Confluent Mv1Lu cells were scratched and allowed to migrate for 6 h. Pictures show the immunostaining with specific antibodies against phospho-FAK (Tyr 925) and paxillin. Co-staining with Hoechst-33258 was used to reveal nuclei. P-FAK: green. Paxillin: red. Nuclei: blue. (**c**) Pictures and graphs represent a colocalization analysis performed by Zeiss Efficient Navigation (ZEN) software. Colocalized pixels are highlighted in white at the immunostaining images. Graphs are dot plots representing both p-FAK and paxillin intensities in terms of number of pixels: p-FAK pixels on Y axis and paxillin pixels on X axis. The 3 quadrant (upper right) represents overlapped (common) pixels between both proteins. (**d**) Pictures of each condition (two) were divided in three horizontally distributed sectors. Pearson’s correlation coefficient in each sector of each condition was calculated by the average pixel intensity of p-FAK and paxillin overlapped pixels. The plot represents Pearson’s correlation values for each condition, each dot representing the value obtained in one sector. Asterisks indicate statistically significant differences between conditions according to one-way ANOVA statistical analysis: (*p < 0.05, **p < 0.005, ***p < 0.001 and ****p < 0.0001). This experiment was repeated at least three times. Representative images are shown. Scale bar indicates 10 µm.

Analyses of various FAK mutants have revealed that FAK targeting to FA is important in the regulation of its activity^51^. Previously, we showed that OA produced an activation of FAK at residue Tyr 925 (see Figs. 2, 4) in MDA-MB-231 cells. Western blot results in Mv1Lu cells confirmed that OA caused a strong FAK phosphorylation on Tyr 925, similarly to EGF, but at a different timing (Fig. 7a). Thus, we decided to study the OA effect on phospho-Tyr 925 FAK subcellular localization at wound-edge migrating cells. As a consequence of wounding, in control cells, we observed a p-FAK sharp and pointed pattern located at FA 6 h after wound scratch (Fig. 7b). Strikingly, upon OA treatment (6 h), the signal increased both at FA and cytosol, thus suggesting the mobilization of the active (phospho-Tyr 925) form of the protein (Fig. 7b). The treatment with EGF produced a similar effect on the increased signal of activated FAK to FA with much less presence to the cytosol. Additionally, the stimulation with OA induced the phosphorylation of paxillin on Ser 178 by JNK (see Supplemental Fig. 4a,b). This phosphorylation supports the association of paxillin with FAK^50^. Strikingly, we noticed a strong merge of p-FAK (Tyr 925) and paxillin in OA-stimulated cells, indicating the localization of active FAK at FAs (Fig. 7c; Supplemental Fig. 7). Pearson´s correlation showed significant values between paxillin and p-FAK (Tyr 925) upon OA treatment that were not noticeable immediately after wounded cells or control (DMSO-treated) cells (Fig. 7d). OA-like results were obtained upon EGF treatment.

Similarly, in MDA-MB-231, we observed that OA produced the phosphorylation of the FAK that was overlapping with the focal structures revealed by paxillin staining. Pearson’s correlation of the staining of both proteins confirmed that phospho-Tyr 925 activated FAK was localized at the focal structures upon OA stimulation (Supplemental Fig. 8a–c).

Therefore, all these results suggest that OA treatment induces an activation of FAK that occurs mainly at the FAs.

### Effect of inhibitors on the OA activation of FAK

**Figure 8:**
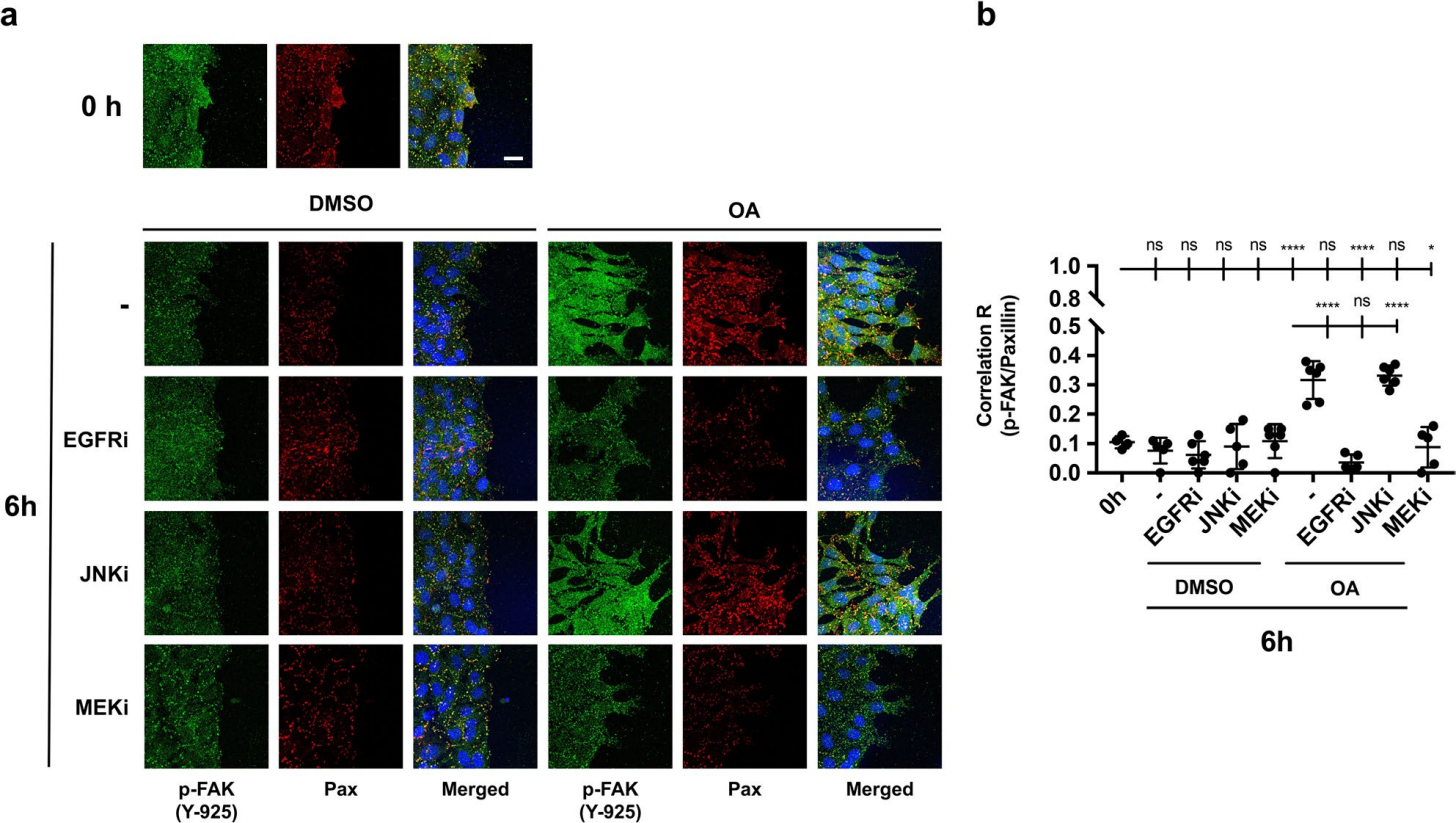
EGFR and MEK inhibitors prevent OA-induced FAK localization at FA in Mv1Lu cells. (**a**) Confluent Mv1Lu cells were scratched and allowed to migrate for 6 h. Cells were treated 30 min before scratch and subsequent OA treatment with specific inhibitors 2,5 µM EGFRi, 50 µM MEKi and 15 µM JNKi. Pictures show the immunostaining with specific antibodies against phospho-FAK (Tyr 925) and paxillin. Co-staining with Hoechst-33258 was used to reveal nuclei. (**b**) The plot represents a colocalization analysis performed by Zeiss Efficient Navigation (ZEN) software. Pictures of each condition (two) were divided in three horizontally distributed sectors. Pearson’s correlation coefficient in each sector of each condition was calculated by the average pixel intensity of p-FAK and paxillin overlapped pixels. The plot represents Pearson’s correlation values for each condition, each dot representing the value obtained in one sector. Asterisks indicate statistically significant differences between conditions according to a one-way ANOVA statistical analysis: (*p < 0.05, **p < 0.005, ***p < 0.001 and ****p < 0.0001). This experiment was repeated at least three times. Representative images are shown. Scale bar indicates 10 µm.

In culture cells, the treatment with OA produced the phosphorylation of FAK at Tyr 925. The use of different inhibitors (EGFRi and MEKi) proved the dependence of this phosphorylation on EGFR and MAPK signaling pathways (see Fig. 4). In fact, the size and the density of FA were affected by EGFRi and MEKi (see Supplemental Fig. 6). To investigate further, we tested the effect of the aforementioned inhibitors on the OA activation of FAK at the wound-edge migrating cells and studied its colocalization with paxillin. Again, the stimulation with OA caused a general phosphorylation at Tyr 925 of FAK, which was evident due to a higher increase in the signal at the FA (Fig. 8a) and its evident colocalization with paxillin (Supplemental Fig. 9). Coherently, this effect was suppressed by the presence of EGFRi and MEKi. However, the presence of JNKi did not affect the activation of FAK measured as localization of phospho-Tyr 925 at the FA as well as a general mobilization of it to the cytosol (Fig. 8a). Pearson´s coefficient, calculated for all samples, clearly indicated the localization of active phosphorylated (Tyr 925) FAK at the FA structures in response to OA, independently of the activation of JNK kinase (Fig. 8b).

All these data suggest a clear OA-stimulated FAK-dependent remodeling of FA in which EGFR and MEK, but not JNK, are involved.

## Discussion

In this paper, we have disclosed more molecular details of OA mode of action on epithelial cells to produce migration and we have studied in depth the effect of OA on critical components of the cell migration machinery. Regarding OA effect on cell migration, it has been reported to inhibit migration on a laryngeal squamous carcinoma cell line (Hep-2) at 30 µM OA concentration in serum-free medium with an additional negative effect on proliferation^11^. Also, on a hepatocellular carcinoma (HepG2) cell line, concentrations of 25 and 50 µM^10^ or 30 µM in the same cell line^9^ have a clear migration inhibitory action. Finally, in HeLa cells, 50 µM of OA has a negative effect on migration and induces apoptosis^8^. However, we previously published that there is a narrow interval of OA concentrations in serum-free medium, 5 µM and 10 µM for Mv1Lu or MDA-MB-231, respectively, that stimulates migration^12^. Above these concentrations, OA inhibits migration in these cell lines^12^. Similarly, the same range of OA concentration for migration stimulation was reported on mouse fibroblasts, 20 µM in serum-free medium, with a migration inhibitory effect above that concentration^6^. This apparent discrepancy could be attributed to the use of different concentrations of OA, although further research should be conducted to possibly stablish differences between the cell lines that may explain the divergence in the results.

Previously, we showed that OA activates c-Jun by phosphorylation in Mv1Lu and MDA-MB-231 cells^12^. C-Jun is a key transcription factor involved in cell migration^52,53^. Active c-Jun plays an important role in the signaling of different growth factors, indeed, conditional mutations of this transcription factor at the epidermis are known to delay wound closure^54^. Our study confirmed that phosphorylation of c-Jun at the wound scratch edge is crucial for the induction of migration of epithelial cells by OA. Remarkably, OA-stimulated c-Jun overexpression and activation only occurred at the nucleus of actively migrating cells, which is consistent with the c-Jun function to promote migration^52,53^ upon OA stimulation. EGF stimulation of cells produces a very extensive c-Jun overexpression and phosphorylation^45,55^. In contrast, the effect of OA was specific to the wound edge of the scratch assay, thus being more precise and avoiding the stimulation of cell migration beyond necessary.

The blockage of upstream molecular signaling by selectively inhibiting MAPKs (MEK1-ERK1/2) or JNK pathways partially inhibited OA-induced migration in Mv1Lu or MDA-MB-231 cells^12^. Moreover, inhibition of EGFR produced a complete inhibitory effect on OA stimulation of migration, suggesting a complete dependence on the activation of EGFR for OA-induced migration^12^. Interestingly enough, the use of EGFRi did not completely suppress phosphorylation of c-Jun at the wound edge. Moreover, OA stimulation of c-Jun phosphorylation measured by western blot was not suppressed by the use of EGFRi. In a previous paper, we focused on the activation of EGFR by OA as the main event inducing migration in both Mv1Lu and MDA-MB-231 cells^12^. However, the evidence presented in this paper suggests a very early stimulation of c-Jun phosphorylation that is independent of the activation of EGFR in both Mv1Lu and MDA-MB-231 cells. Additional pathways converge into c-Jun activation^56,57^; nevertheless, all the c-Jun activation we saw, independent of or dependent on EGFR was mediated by JNK1/2. JNK has been shown to be required for Drosophila dorsal closure^15,16^. Certainly, the use of JNKi completely blocked the phosphorylation of c-Jun. In any case, further research must be carried out in order to reveal the possible pathway that specifically produces the activation of c-Jun, independently of EGFR activation.

C-Jun, the founding member of the AP-1 family, contributes to its own transcriptional upregulation when it is activated by phosphorylation, producing a self-regulating positive feed-back^56^. OA phosphorylation/protein total level increment of c-Jun and phosphorylation of EGFR showed different time dynamics, with EGFR having a later activation compared to c-Jun activation, which suggests a complex or multiple step mechanism for OA activation of EGFR. In this context, transactivation of EGFR is a phenomenon that activates this receptor in the absence of canonical EGFR external stimuli^58^. EGFR transactivation is triggered by G-protein-coupled receptors (GPCRs) and is now recognized as a key mechanism that couples distinct signal transduction pathways to diverse cellular stimuli^58^. It is plausible that EGFR stimulation by OA could be due to receptor transactivation. Indeed, a clue comes from the fact that OA has been identified as a weak agonist for some GPCR receptors^59^, which in turn would activate EGFR^60^ and could also have a direct stimulatory effect on the MAPK signaling pathway^61^. Besides, as shown by Bernabé et al.^12^, the stimulation of EGFR phosphorylation by OA is independent of protein synthesis. Despite all of this, activation of EGFR is clearly a key point in the process of migration induced by OA, because the inhibition of the receptor by PD153035 completely suppresses OA stimulation of migration^12^.

In Mv1Lu cells, the OA-induced phosphorylation of ERK experienced a delay in comparison to c-Jun phosphorylation, and it is in synchrony with the activation of EGFR. This fact suggests that MAPK signaling pathway mainly depends on the OA-activation of EGFR. Unfortunately, in MDA-MB-231, the high overexpression of EGFR^44^, causes a high level of ERK phosphorylation making it impossible to clearly distinguish ERK activation in response to OA.

So far, our results show that, in Mv1Lu and MDA-MB-231 cell lines, JNK and c-Jun OA-activation are independent of EGFR. On the other hand, our results for ERK phosphorylation support an EGFR delayed activation mechanism independent of the mechanism early activating JNK/c-Jun. In any case, MAP kinases seem to be involved in both mechanisms, early and late, because PD98059 (MEK) inhibitor blocks the activation of both EGFR and JNK/c-Jun. This way of behaving would suggest a possible feedback mechanism between the MAPK pathway and the activation mechanism that implicates both EGFR and putative GPCR. This crosstalk mechanism between both receptors has been associated with the activation of Src^61^, but it is worth exploring whether there could be a similar crosstalk for other kinases such as ERK1/2. In the wounding of corneal cells, the activation of EGFR is mediated by a shedding mechanism related to GPCR receptors^62^. Strikingly, in this system, the inhibition of ERK1/2 phosphorylation by the use of PD98059 caused a halt in the activation of EGFR, thus suggesting that, in addition to functioning as an EGFR downstream effector, ERK1/2 also mediates EGFR transactivation to a variety of stimuli^62^. Similarly, in both MDA-MB-231 and Mv1Lu cells, the use of PD98059 upon OA stimulation caused a strong inhibitory effect on EGFR activation and c-Jun phosphorylation. This suggests that, upon stimulation with OA, a similar MEK-involved crosstalk mechanism could be at work for the transactivation of EGFR. In fact, in Mv1Lu cells, a minute stimulation of ERK could be seen 1 h after OA stimulation (see Fig. 3).

At in vitro wound assays, a close look at the wound migrating edge showed morphological variations in migrating cells at the wound edge compared to cells placed in the rear. These morphological differences consist in the development of cytoplasmic protrusions such as lamellipodia and ruffles^19^. Indeed, the presence of OA increased the number of lamellipodia and ruffles at the cells at the edge of the wound. In our case, the stimulation with OA also modified the intensity and definition of actin microfilaments there. In fact, actin depolymerization and debranching happens during migration facilitating the dynamic remodeling of the actin network and the cyclic extension and retraction of lamellipodia^20^. So, the observed changes were suggestive of an active migratory status of the cells^19,20^. Cell migration, a very well-coordinated process, requires the continuous formation of focal complexes (FC) that mature into FAs^19,20^. Paxillin is a fundamental component for FA functioning because it is able to integrate multiple signaling inputs^24^. By providing a structural scaffold, paxillin facilitates the synchronized binding of different protein components, particularly signaling molecules^63^. Our results showed a differential organization of FA, revealed by paxillin staining, in response to OA. Certainly, OA caused an increased density but a decreased size of the foci. The phosphorylation of paxillin Ser 178 by JNK produced FA increased dynamics and cell motility^28^. OA stimulation produced a time phosphorylation on this residue that correlated well with JNK activation. However, in response to OA, neither the size nor the density of FA were affected by the presence of this inhibitor (JNKi), which suggests that there might be other means of focal adhesion arrangements in response to OA, independently of JNK activation. Further research is needed to stablish the particular contribution of paxillin Ser 178 phosphorylation to the migration induced by OA.

Coordination and integration of growth-factor signaling and FA remodeling is facilitated by the close physical proximity of key signaling molecules. This is the case for EGFR and FAK^64^. FAK Tyr 925 can be phosphorylated as a consequence of cell integrin assembling or growth factor stimulation^31,36^. Interestingly, OA treatment produced the phosphorylation of EGFR and thus of FAK with a very similar timing outcome. Indeed, FAK activation was completely suppressed by EGFRi. Additionally, FAK Tyr 925 phosphorylation was also suppressed by MEKi, indicating the direct involvement of EGFR via MEK in the activation of FAK in response to OA. So, regarding FA remodeling and in contrast to JNKi, the presence of EGFRi or MEKi produced bigger and consolidated FAs, thus supporting previous findings indicating the need of an EGFR/MEK active signaling for a successful OA-driven migration^12^.

The use of different functional mutants of FAK has revealed that FAK localization to FAs is important for the regulation of FA dynamization^51^. Hence, increased FAK localization to FAs raises its turnover at the protrusion front^34^. Our results show that active FAK co-localized with paxillin in response to OA, thus indicating that an active remodeling of FAs was taking place in response to the triterpenoid. Active FAK localization at FAs was dependent on the activation of EGFR because it was inhibited by EGFRi. Also, the participation of active MEK was required as the use of MEKi also prevented Tyr 925 phosphorylated FAK localization at FAs. These data confirmed an OA responsive axis EGFR/MEK/ERK/FAK as a motor for FA dynamization^49^. Additionally, mobilization of phospho-Tyr 925 FAK to the cytosol indicates an intense remodeling of FAs. Certainly, active migration requires a continuous remodeling of FAs^27^. The absence of inhibitory activity on the FA localization of active FAK in the presence of JNK reinforces the idea of an independent but complementary promigratory event. Such promigratory event, although not required for FA managing, may be involved in other aspects of cell migration. So, even though active FAK mobilization to FAs did not seem to be affected by JNKi, OA-stimulated cell migration is partially inhibited^12^.

In this paper, we have further dissected the molecular mechanisms underlying the effect of OA on cell migration. Our results showed that OA induces different and interconnected signaling pathways. Given the powerful effect of OA on cell-migration, the need for additional understanding of those mechanisms seems relevant and very pertinent. Finally, these findings could provide an interesting mode of action for OA that is worth investigating in other related molecules.

## Materials and methods

### Preparation of oleanolic acid

Oleanolic acid (purity > 97%) (Merck, Darmstadt, Germany) was dissolved in dimethyl sulfoxide (DMSO) to a 25 mM concentration. Final assay concentrations are indicated in each experiment. DMSO concentration in all assays never exceeded 1% to avoid cytotoxic effects. A DMSO control condition was performed in each experiment.

### Cell culture

Mink Lung Epithelial (Mv1Lu)^38,40,65^ cells were grown in Eagle's Minimum Essential Medium (EMEM) (Biowest, Nuaillé, France). Human Mammary Gland cells (MDA-MB-231)^41–43^ were grown in Dulbecco's Modified Eagle Medium (DMEM) (Biowest, Nuaillé, France). Both mediums were supplemented with 10% Fetal Bovine Serum (FBS, Gibco, Thermo Fisher Scientific, Rockford, IL, USA), 1% Penicillin/Streptomycin and 1% L Glutamine (both from Biowest, Nuaillé, France). Mv1Lu and MDA-MB-231 cells were incubated in a humidified atmosphere at 37 °C, with 5% CO_2_ and 7.5% CO_2_, respectively.

### Wound healing scratch assay

Mv1Lu or MDA-MB-231 cells were seeded in 24-well plates until they reached 100% confluence, using the appropriate medium for each line with all supplements. At that time, medium was changed to FBS-free medium for 24 h. At the initial time (T0), a cross-shaped scratch was made on the cell monolayer using a sterile p-200 µl pipette tip. After replacing FBS-free culture medium to wash out released cells, OA (indicated concentration at each experiment) or an equivalent volume of DMSO was added. Additionally, EGFR pharmacological inhibitor PD153035 (EGFRi) 2.5 µM, was added to OA sample. After a 24-h incubation period, cells were fixed with 4% formaldehyde (Applichem GmbH, Darmstadt, Germany) in PBS (Biowest, Nuaillé, France) for 10 min. Finally, well plates were washed twice with PBS. Pictures were taken at 10× magnification using an optical microscope equipped with a digital camera (Motic Optic AE31, Motic Spain, Barcelona, Spain). To quantify cell migration, the areas of the gaps in the wounds were measured by ImageJ software. The initial cell‐free surface was subtracted from the endpoint cell‐free surface and plotted in a graph^66^.

### Cell-front migration assay, subcellular localization assay

Mv1Lu or MDA-MB-231 cells were grown until they reached confluence on round glass-coverslips using appropriate medium with 10% FBS. At that point, cells were washed and the medium was replaced by FBS-free medium for 24 h. After this, the established epithelium was scratched using a razor blade, making an artificial wound with enough space to allow cells to migrate. The new wound was set as time 0 of the experiment and then OA, DMSO or 10 ng/ml epidermal growth factor (EGF, Sigma-Aldrich, St Louis, MO, USA) was added to the medium. When using specific inhibitors, cells were treated 30 min before OA stimulation with the following pharmacological inhibitors: 2.5 µM PD153035 (Epidermal Growth Factor inhibitor, EGFRi)^68^, 50 µM PD098059 (Mitogen-activated protein kinase kinase inhibitor, MEKi)^69^ and 15 µM SP600125 (c-Jun N-terminal kinase inhibitor, JNKi)^70^. After the indicated times of incubation, coverslips were fixed with 4% formaldehyde (Applichem GmbH, Darmstadt, Germany) in PBS (Biowest, Nuaillé, France) for 10 min. After two washes with PBS, cells were permeabilized with 0.3% Triton X-100 (Sigma-Aldrich, St Louis, MO, USA) in PBS for 10 min. Immunostaining was performed first by blocking cells for 30 min at room temperature in PBS solution with 10% FBS, 5% skim milk (Beckton Dickinson, Franklin Lakes, NJ, USA), 0.3% bovine serum albumin (BSA, Sigma-Aldrich, St Louis, MO, USA) and 0.1% Triton X-100. After blocking the cells, they were incubated for 1 h at room temperature with anti-paxillin, anti-c-Jun or anti-phospho-FAK antibodies diluted in blocking solution, without skim milk. Appropriate fluorescent-labelled secondary antibodies were co-incubated for 30 min with Alexa Fluor 594 conjugated phalloidin (Molecular Probes, Thermo Fisher Scientific, Waltham, MA, USA) and Hoechst-33258 (Fluka, Biochemika, Sigma-Aldrich, St Louis, MO, USA) to reveal actin cytoskeleton and nuclei, respectively. Finally, samples were examined and representative images were taken with a confocal microscope (LSM 510 META from ZEISS, Jena, Germany). Images were obtained by using Zeiss Efficient Navigation (ZEN) interface software (ZEISS, Jena, Germany). When a wider view of the migration front was required, especially in c-Jun staining (indicated in figures), four linked fields were acquired by the “Tile scan” ZEN tool. Subsequently, tile scans fluorescent signals were converted to a linear mode and covered with a full data range using the Rainbow look up table (Rainbow LUT) in ImageJ software. In order to quantify c-Jun and phospho-c-Jun levels in immunofluorescence pictures, the image was analyzed and quantified by ImageJ software. For this purpose, pictures in 8-bit three-channel format (Red, Green, Blue, RGB) were divided into three separate color channels (three pictures). By using blue channel picture (Hoechst staining), regions of interest (ROIs) were stablished to define each nucleus, creating as many ROIs masks as nuclei in the image. Then, by overlapping these masks onto the corresponding green channel picture (c-Jun/p-c-Jun staining), we calculated the green intensity value of each nucleus (ROI). The quantified signal of each nucleus was used as a replicate to obtain c-Jun/p-c-Jun intensity data in each of the conditions performed. On the other hand, “Z stack” ZEN feature was used when deep cytoskeleton structure observance was required, taking a proper number of pictures along the Z axis. Focal adhesion (FA) quantifications were performed as described in Horzum et al.^67^ by using CLAHE and Log3D macros for ImageJ. Briefly, focal adhesions were quantified from paxillin stained acquired pictures. We used three different images for each condition. Specifically, cell filopodia were selected as regions of interest (ROIs) and the resulting areas (containing FAs) were considered for further analysis. A number of five filopodia were taken into account from each picture. Then, the number of FAs were calculated in each filopodia by using the previously mentioned macros. The obtained number was divided by the total filopodia area to determine FA density. In parallel, the size of each FA was measured using the macros mentioned above. Finally, FA average size was calculated for each treatment. For colocalization determination of paxillin and p-FAK, ZEN interface software was used. The analysis was performed by dividing two pictures (replicates) of each experimental condition into three equal regions of interest (ROIs) including front and back of the edge. Then, overlapped pixels between channel 1 signal (paxillin) and channel 2 signal (p-FAK) were marked for a Pearson’s correlation coefficient determination included in ZEN software (ZEISS, Jena, Germany).

### Western blot

Mv1Lu or MDA-MB-231 cells were seeded and allowed to reach 60% confluence in 10-cm diameter plates. At that time, culture medium containing 10% FBS was replaced by an FBS-free medium, incubating the cells for a 24-h period. Serum-deprived cells were treated with OA, DMSO or 10 ng/ml EGF. When using specific inhibitors, cells were treated 30 min before OA stimulation with the following pharmacological inhibitors: 2.5 µM PD153035 (Epidermal Growth Factor inhibitor, EGFRi)^68^, 50 µM PD098059 (Mitogen-activated protein kinase kinase inhibitor, MEKi)^69^ and 15 µM SP600125 (c-Jun N-terminal kinase inhibitor, JNKi)^70^. After time incubations, cells were harvested, washed twice with ice cold PBS and lysed with 20 mM TRIS pH 7.5, 150 mM NaCl, 1 mM EDTA, 1.2 mM MgCl_2_, 0.5% Nonidet p40, 1 mM DTT, 25 mM NaF and 25 mM beta-glycerophosphate supplemented with phosphatase inhibitors (I and II) and protease inhibitors (all from Sigma-Aldrich, St Louis, MO, USA). Total protein amount of all extracts was measured and normalized by Bradford assay^71^ (Sigma-Aldrich, St Louis, MO, USA). The extracts were analyzed by SDS-PAGE followed by Western blot (WB) using the indicated antibodies, revealed by using Horseradish peroxidase substrate (ECL) (GE Healthcare, GE, Little Chalfont, United Kingdom) and images were taken with a Chemidoc XRS1 (Bio-Rad, Hercules, CA, USA). For protein quantification, western pictures in 8-bit format were processed in ImageJ software. Subsequently, in all western-blot pictures, a lane was stablished for each of the samples. In each lane, the band was selected according to the appropriate size (kDa) of the protein of interest. In general, and for the phosphorylated version of each interest protein, each band’s intensity peak was plotted and the area under the plot was measured using “Wand (tracing) tool” of ImageJ to obtain the intensity value. To normalize the data, obtained intensity values were referred to obtained intensity values of either the unphosphorylated form of the protein or a loading control protein such as β-actin if the unphosphorylated form was undetectable (non-available antibody for detecting the unphosphorylated form).

### Antibodies

The following commercial primary antibodies were used: anti-phospho-ERK1/2, anti-ERK, anti-JNK1/2, anti-phospho-JNK1/2, and anti-phospho-c-Jun (all from Cell Signaling Technology, Danvers, MA, USA); anti-phospho-EGFR, anti-EGFR, anti-FAK (all from Thermo Fisher Scientific, Rockford, IL, USA); anti-phospho-FAK (Abcam, Cambridge, MA, USA); anti-paxillin and anti-c-Jun (Santa Cruz Biotechnology, Heidelberg, Germany); anti-phospho-paxillin Ser 178 (Antibodies-online GmbH, Aachen, Germany); and anti-β-actin (Sigma-Aldrich, St Louis, MO, USA). Secondary antibodies were anti-rabbit IgG Horseradish peroxidase linked F(ab')2 I fragment (from donkey) (GE Healthcare, GE, Little Chalfont, United Kingdom), anti-mouse IgG_1_ (BD Pharmingen, Beckton Dickinson, Franklin Lakes, NJ, USA); Alexa Fluor 488 conjugated anti-rabbit (from donkey), Alexa Fluor 488 conjugated anti-mouse (from donkey) and Alexa Fluor 594 conjugated anti-mouse (from donkey) (all from Thermo Fisher Scientific, Rockford, IL, USA).

### Statistical analysis

All the collected data were analyzed using Graph Pad Prism 7 software. In every analysis, classic statistical parameters were calculated and statistical tests were performed with a 95% confidence interval, consequently, p values lower than 0.05 were considered to be statistically significant. At the figure legends, the asterisks denote statistically significant difference between treatments (*p < 0.05, **p < 0.005, ***p < 0.001 and ****p < 0.0001). Data of intensity values, collected from Western-blots, were analyzed by a one-way ANOVA test, comparing the mean of each condition with the mean of every other condition. Then, a Tukey’s multiple comparisons test was performed. Data of intensity values obtained from c-Jun and p-c-Jun nuclei quantifications were analyzed by a two-way ANOVA test. Specifically, a Tukey’s multiple comparisons test was performed, comparing the mean of each condition with the mean of every other condition; however, each comparison was made within each picture sector. In this way, same picture sectors were compared (for example, sector 1, S1) between different conditions. On the other hand, to see differences between sectors from the same condition a one-way ANOVA was performed, as described elsewhere. Data of colocalization Pearson’s coefficient in the immunocytochemistry assays were analyzed with a one-way ANOVA test, comparing the mean of each condition with the mean of every other condition. Then, a Tukey’s multiple comparisons test was performed.

## Supplementary Information

Supplementary Figure 1.

Supplementary Figure 2.

Supplementary Figure 3.

Supplementary Figure 4.

Supplementary Figure 5.

Supplementary Figure 6.

Supplementary Figure 7.

Supplementary Figure 8.

Supplementary Figure 9.

Supplementary Figure 10.

Supplementary Figure 11.

Supplementary Figure 12.

Supplementary Figure 13.

Supplementary Figure 14.

Supplementary Figure 15.

## Data Availability

In order to clarify Western-blot data, we displayed cropped blots of each Figure in the paper as Supplementary Figures (from Supp. Figures 10–15). These Figures contain original blots of each protein assayed with molecular weight markers for a better identification.
