# Supplementary figures and images for: Oleanolic acid stimulation of cell migration involves a biphasic signaling mechanism
Source: Sci Rep. 2022 Sep 5;12:15065. doi: 10.1038/s41598-022-17553-w (PMC9445025; doi:10.1038/s41598-022-17553-w)

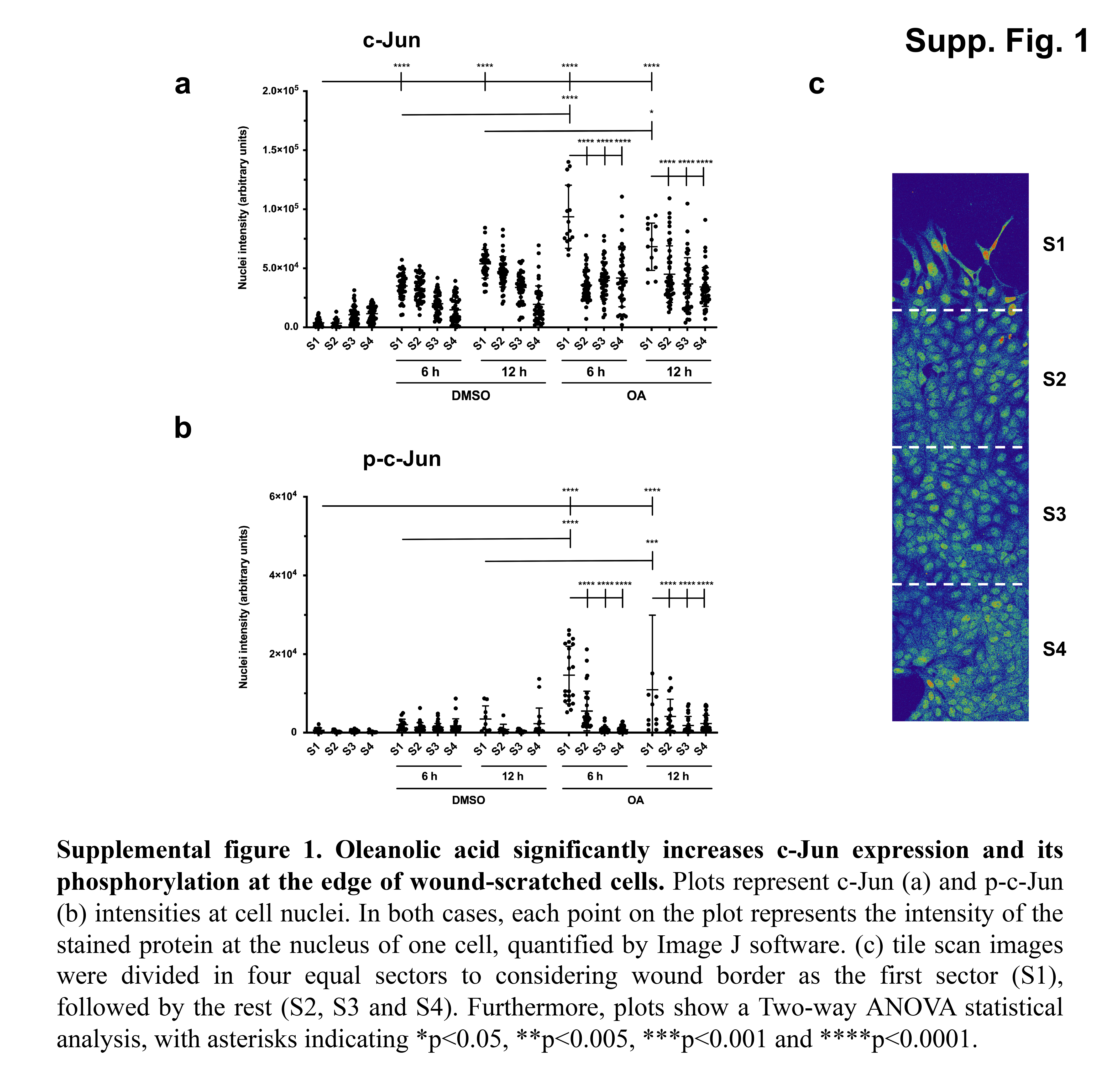

Supplement: Supplementary file 1 — Supplementary Figure 1. [file 41598_2022_17553_MOESM1_ESM.tif]

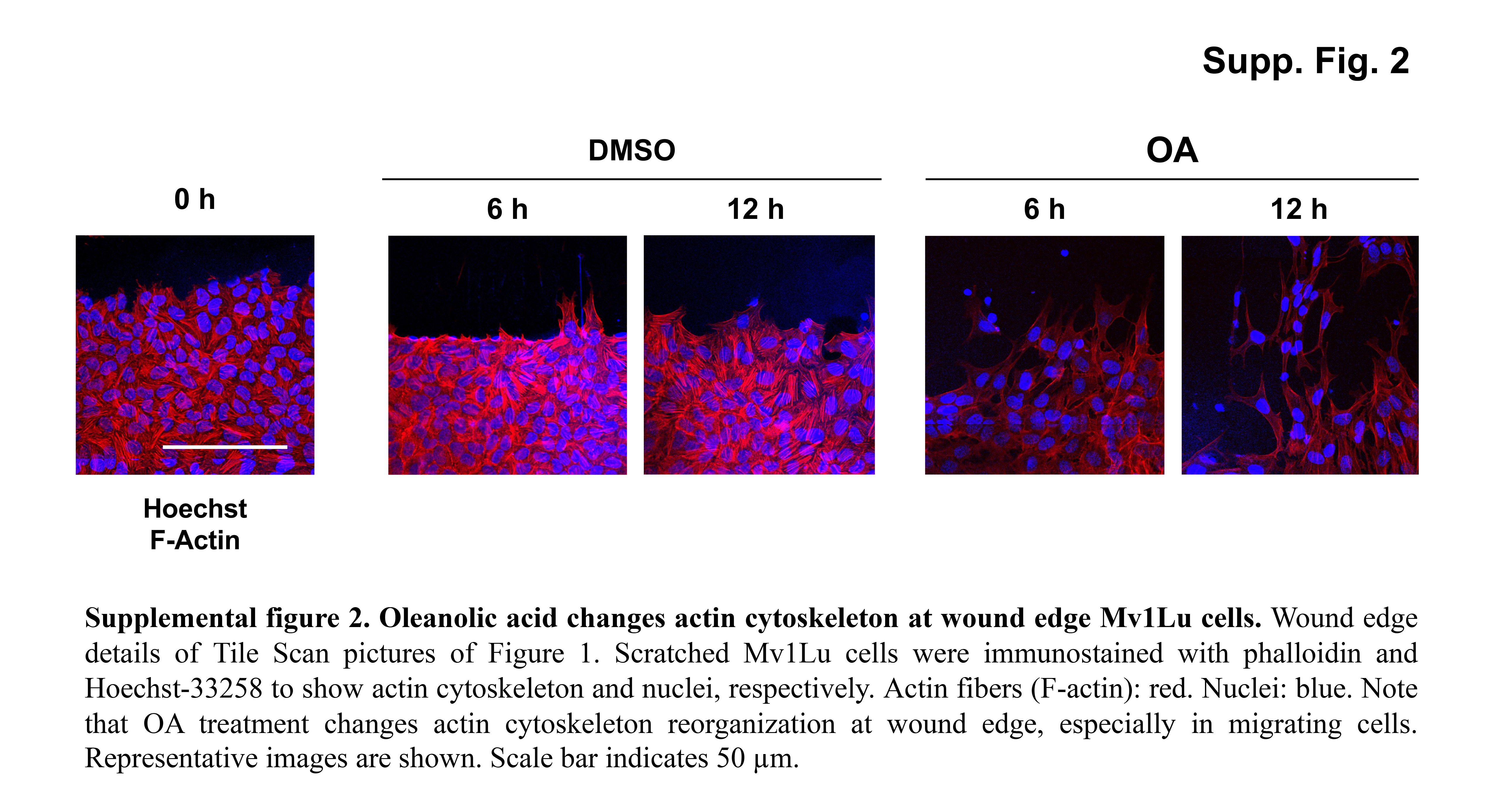

Supplement: Supplementary file 2 — Supplementary Figure 2. [file 41598_2022_17553_MOESM2_ESM.tif]

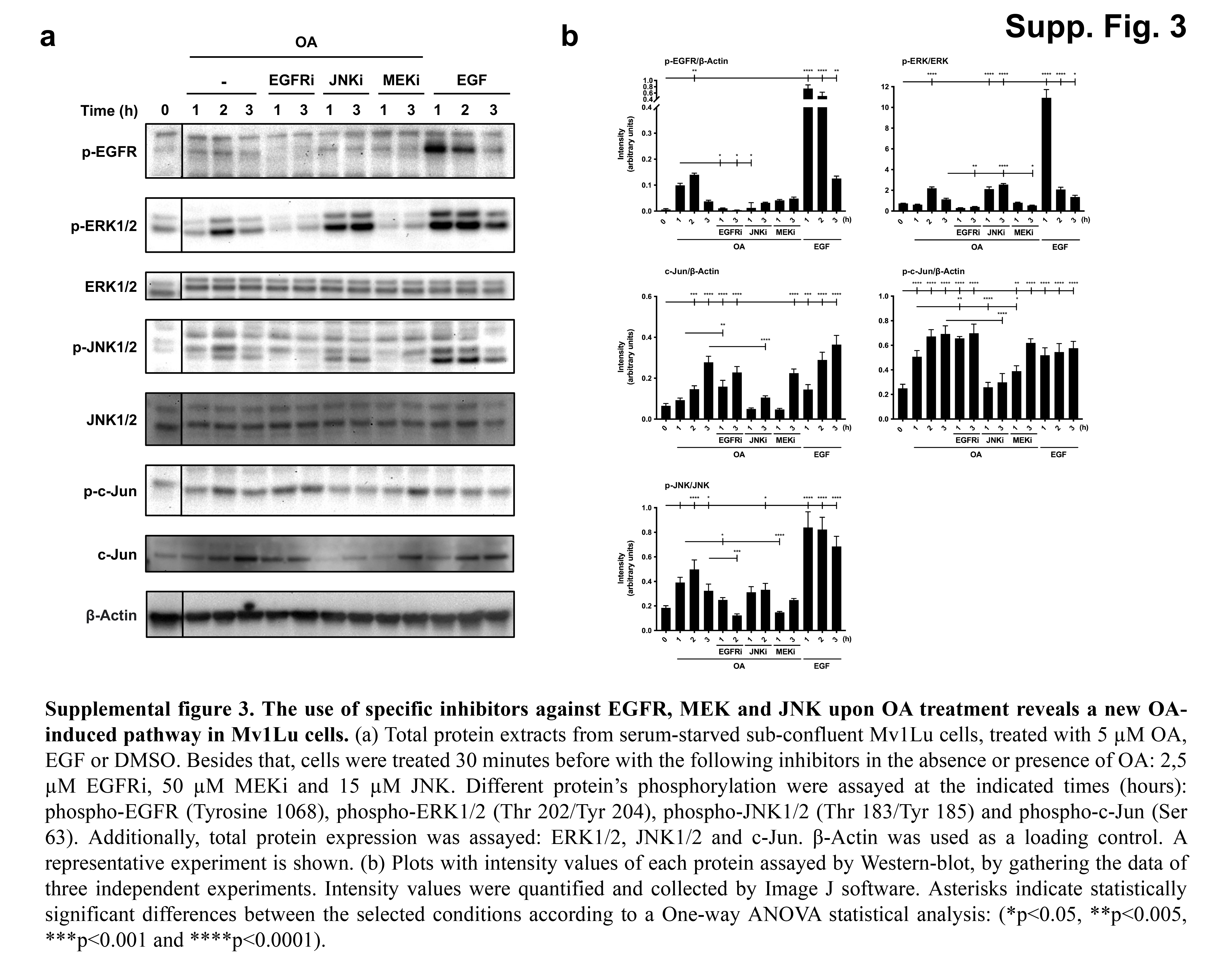

Supplement: Supplementary file 3 — Supplementary Figure 3. [file 41598_2022_17553_MOESM3_ESM.tif]

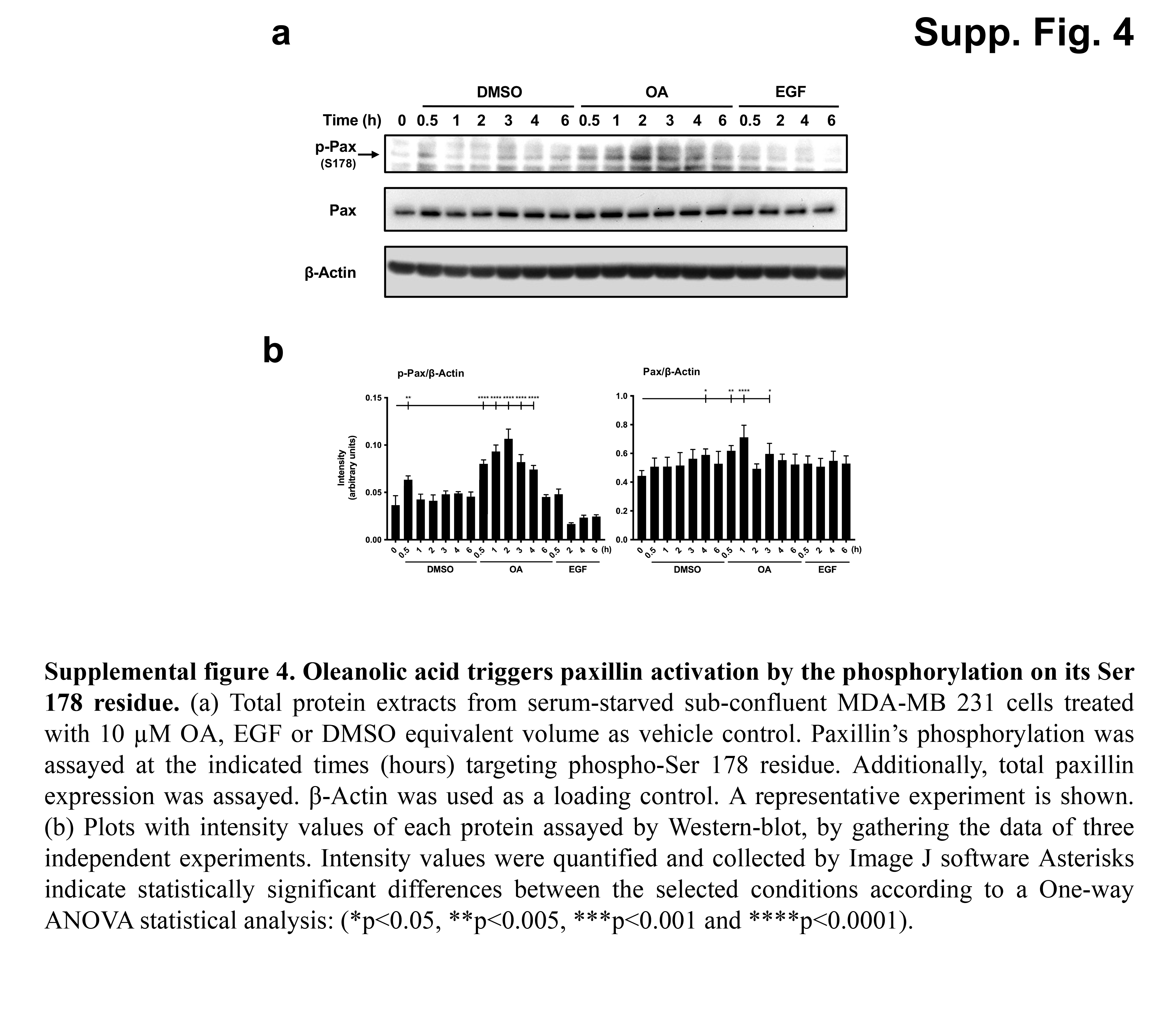

Supplement: Supplementary file 4 — Supplementary Figure 4. [file 41598_2022_17553_MOESM4_ESM.tif]

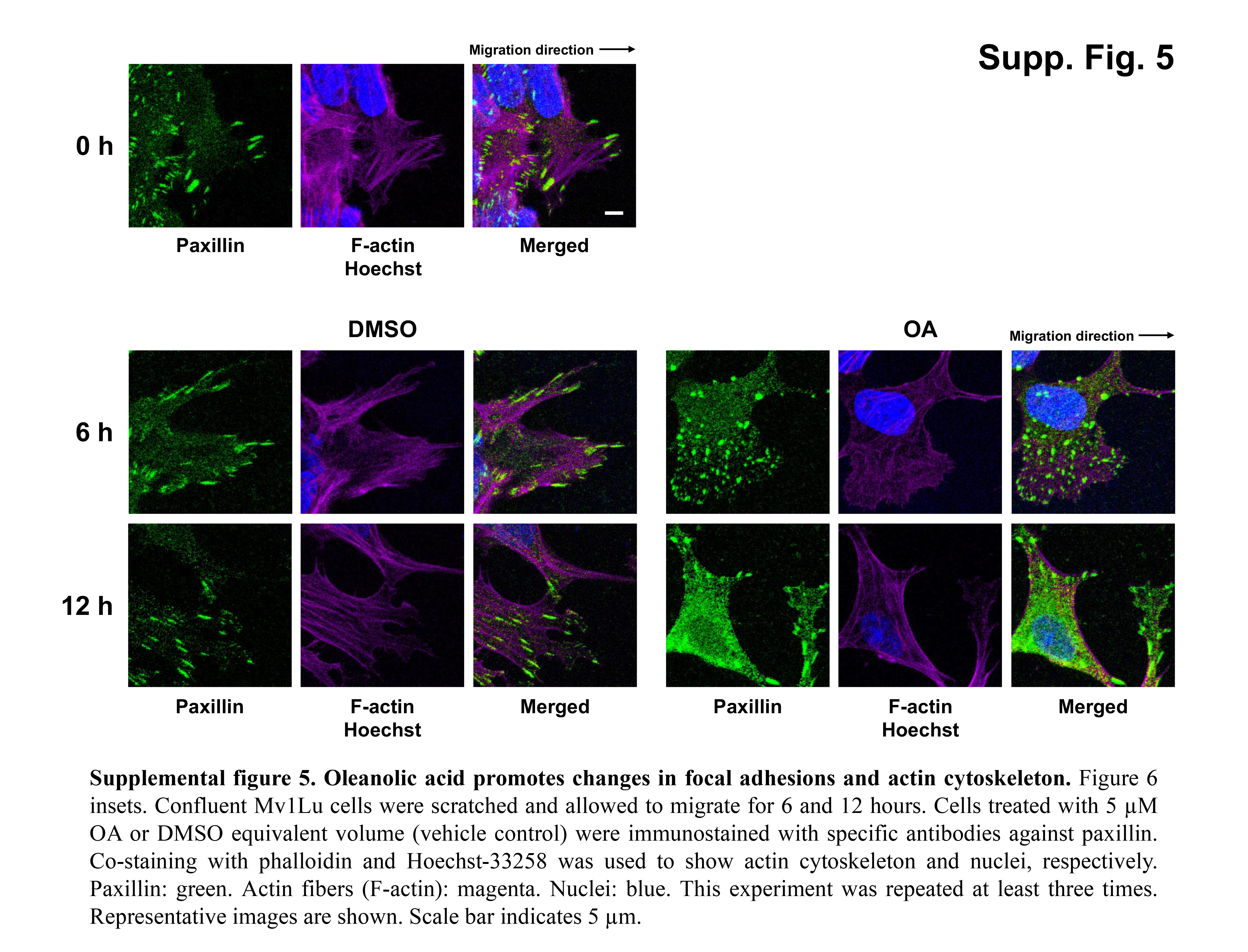

Supplement: Supplementary file 5 — Supplementary Figure 5. [file 41598_2022_17553_MOESM5_ESM.tif]

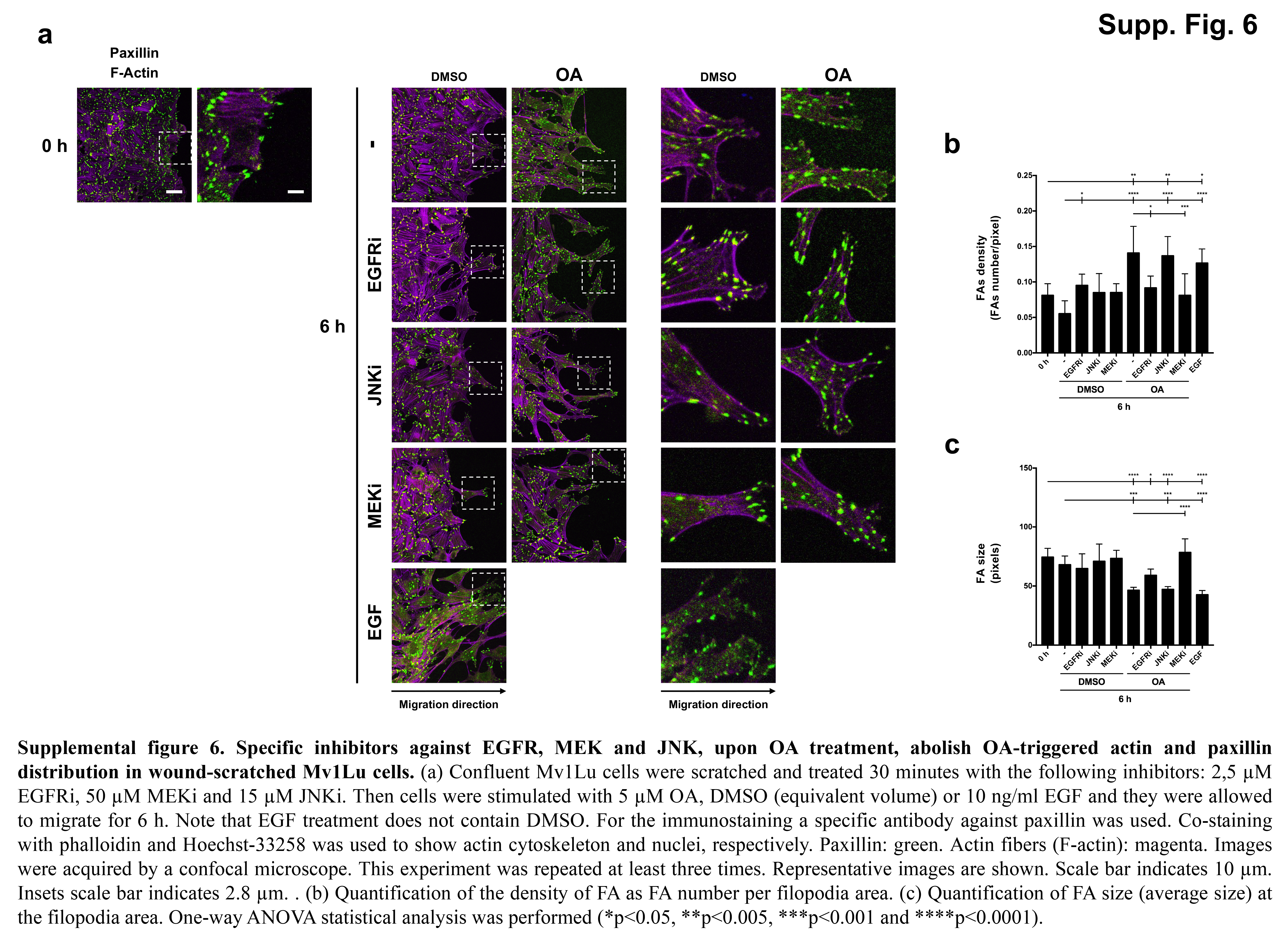

Supplement: Supplementary file 6 — Supplementary Figure 6. [file 41598_2022_17553_MOESM6_ESM.tif]

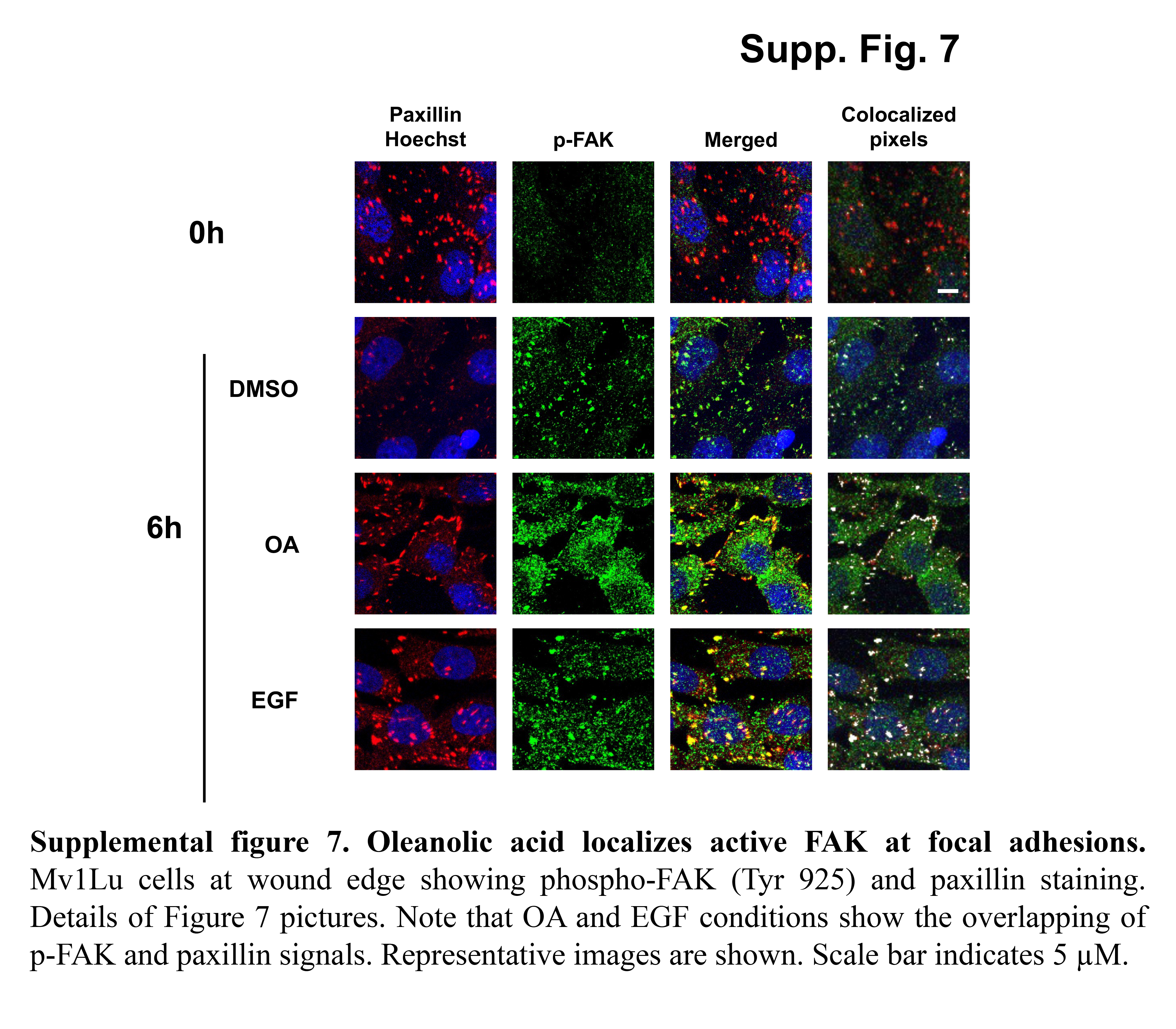

Supplement: Supplementary file 7 — Supplementary Figure 7. [file 41598_2022_17553_MOESM7_ESM.tif]

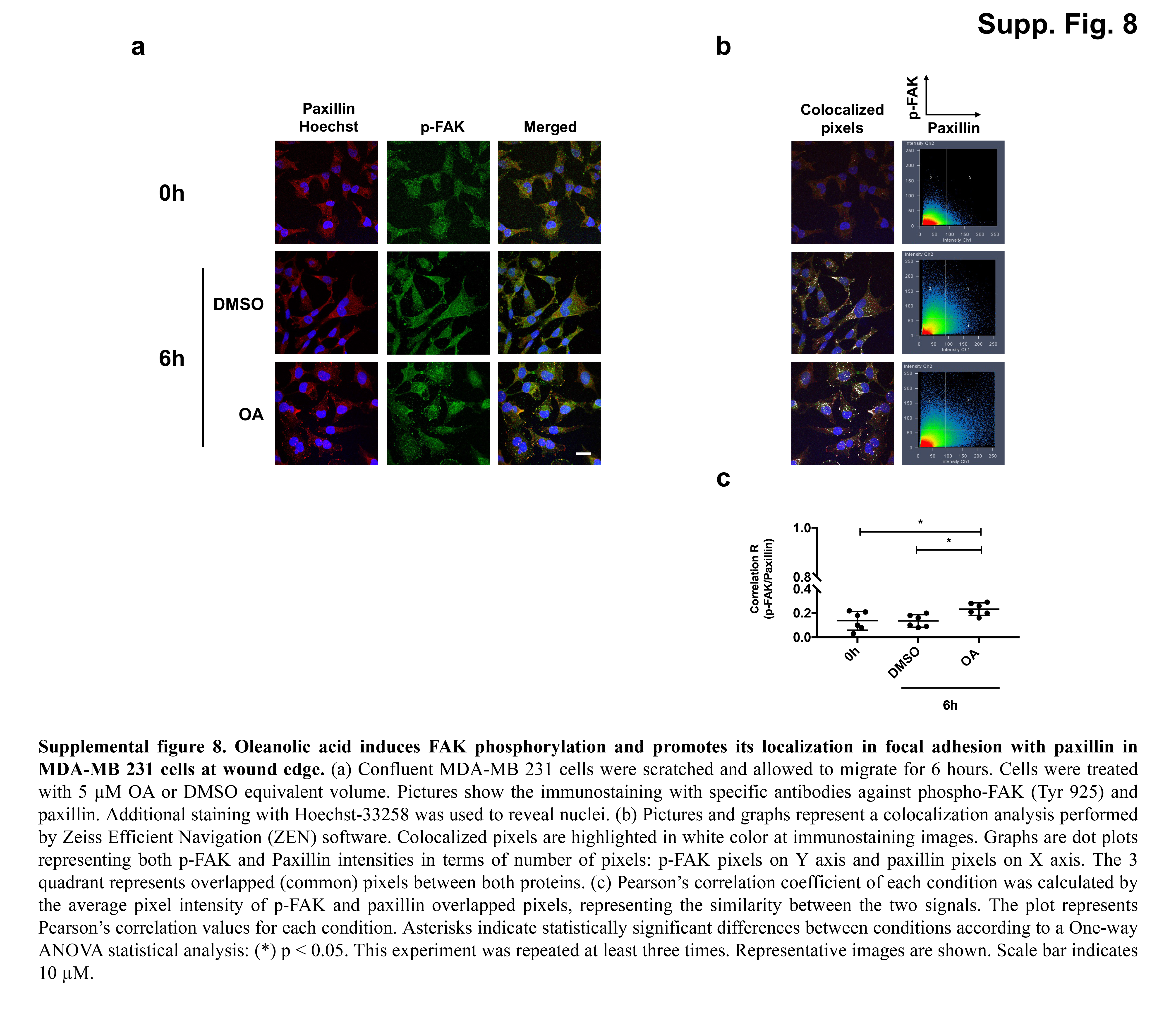

Supplement: Supplementary file 8 — Supplementary Figure 8. [file 41598_2022_17553_MOESM8_ESM.tif]

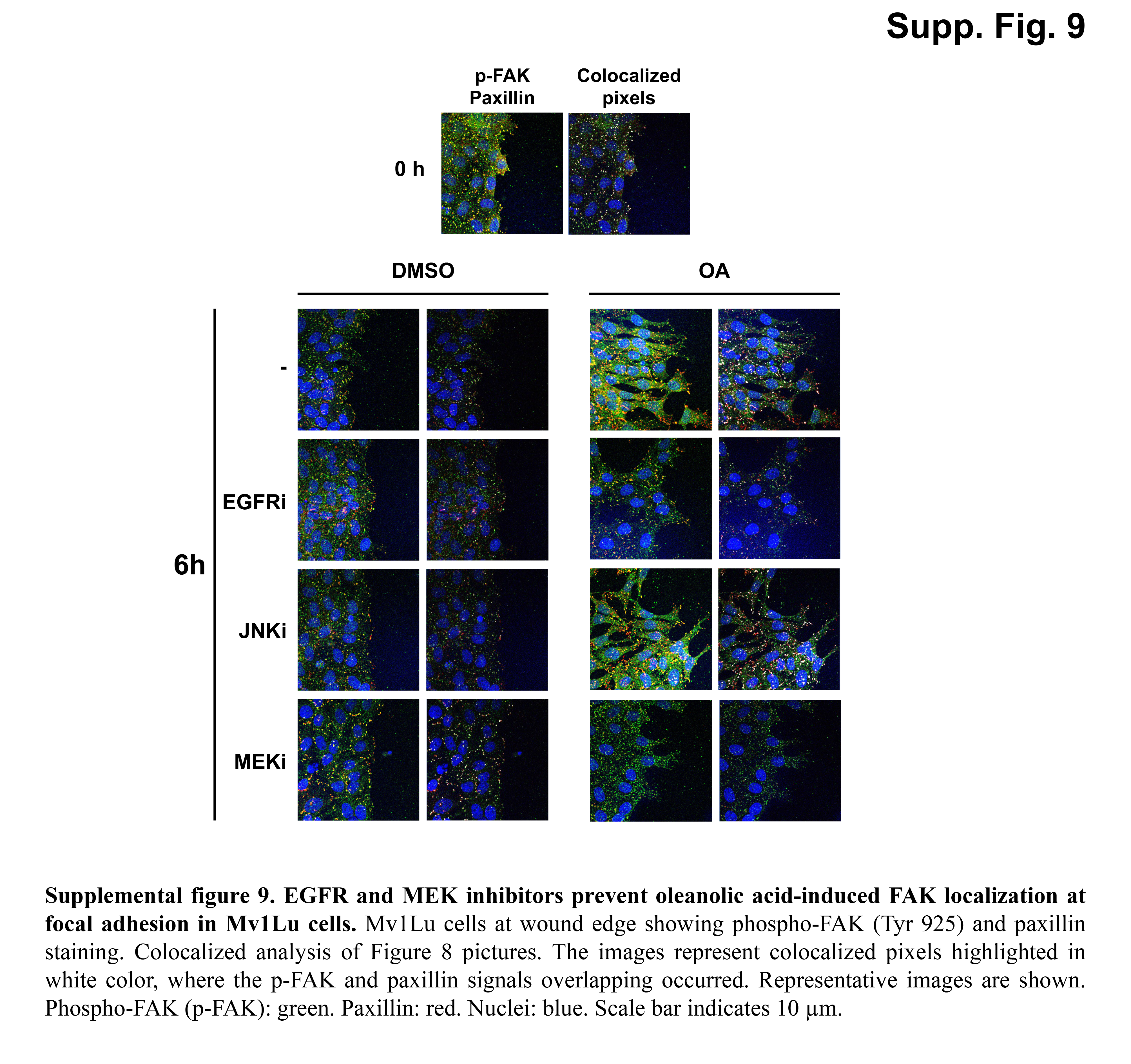

Supplement: Supplementary file 9 — Supplementary Figure 9. [file 41598_2022_17553_MOESM9_ESM.tif]
