# Supplementary material for: Oleanolic acid stimulation of cell migration involves a biphasic signaling mechanism
Source: Sci Rep. 2022 Sep 5;12:15065. doi: 10.1038/s41598-022-17553-w (PMC9445025; doi:10.1038/s41598-022-17553-w)
Supplement: Supplementary file 10 — Supplementary Figure 10. [file 41598_2022_17553_MOESM10_ESM.pdf]

Supp. Fig. 10

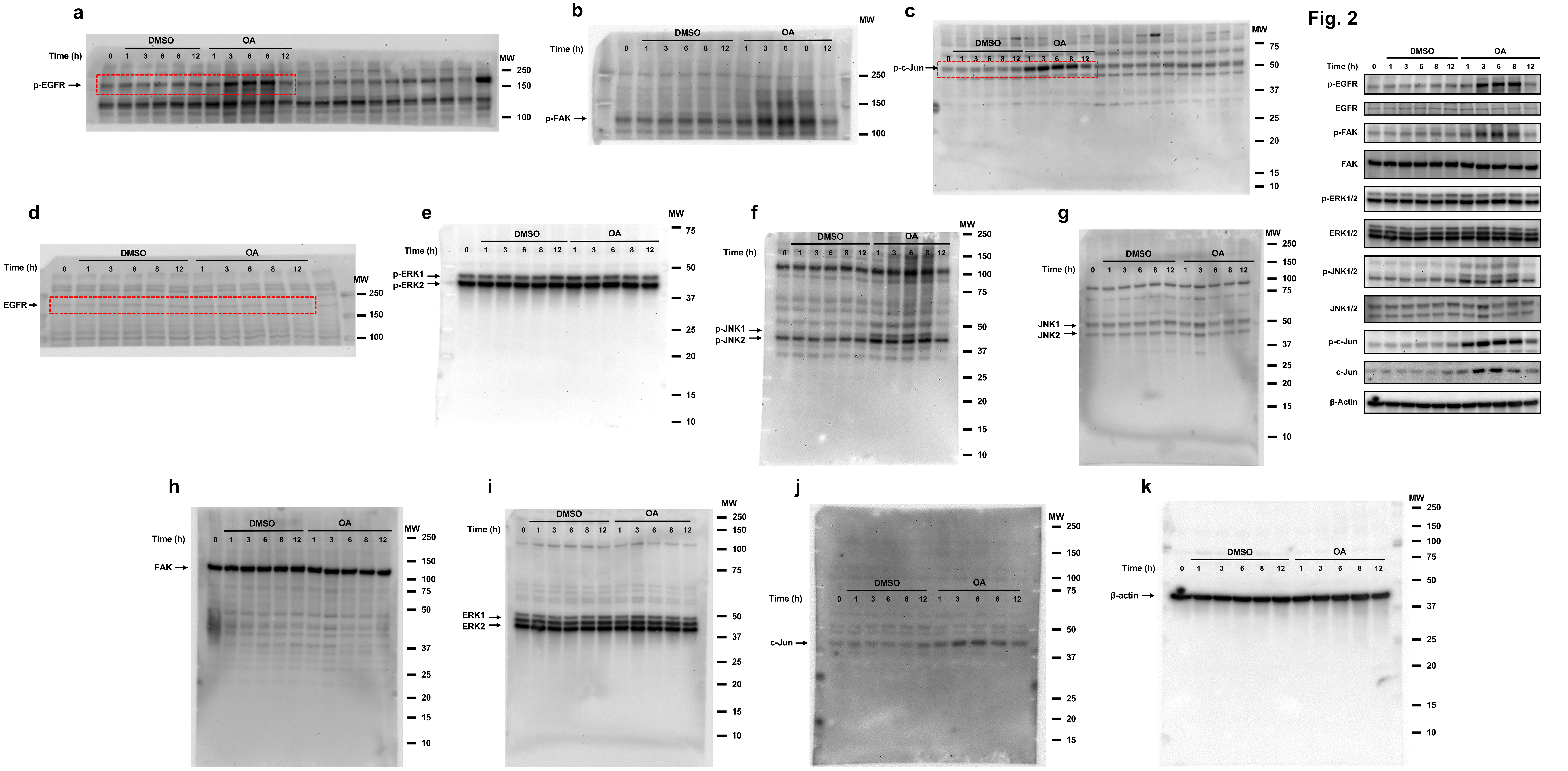

Supplemental Figure 10. Full-length blots corresponding to crops showed in Fig 2. (a) Tyr 1068 Phosphorylated-EGFR. (b) Tyr 925 Phosphorylated-FAK. (c) Ser 63 Phosphorylated c-Jun. (d) EGFR. (e) Thr 202/Tyr 204 Phosphorylated ERK. (f) Thr 183/Tyr 185 Phosphorylated JNK. (g) JNK1/2. (h) FAK. (i) ERK1/2. (j) c-Jun. (k) Beta-actin loading. Dashed red rectangle indicates the portion of the blot that was used in the figure.
