# Supplementary material for: Oleanolic acid stimulation of cell migration involves a biphasic signaling mechanism
Source: Sci Rep. 2022 Sep 5;12:15065. doi: 10.1038/s41598-022-17553-w (PMC9445025; doi:10.1038/s41598-022-17553-w)
Supplement: Supplementary file 11 — Supplementary Figure 11. [file 41598_2022_17553_MOESM11_ESM.pdf]

Supp. Fig. 11

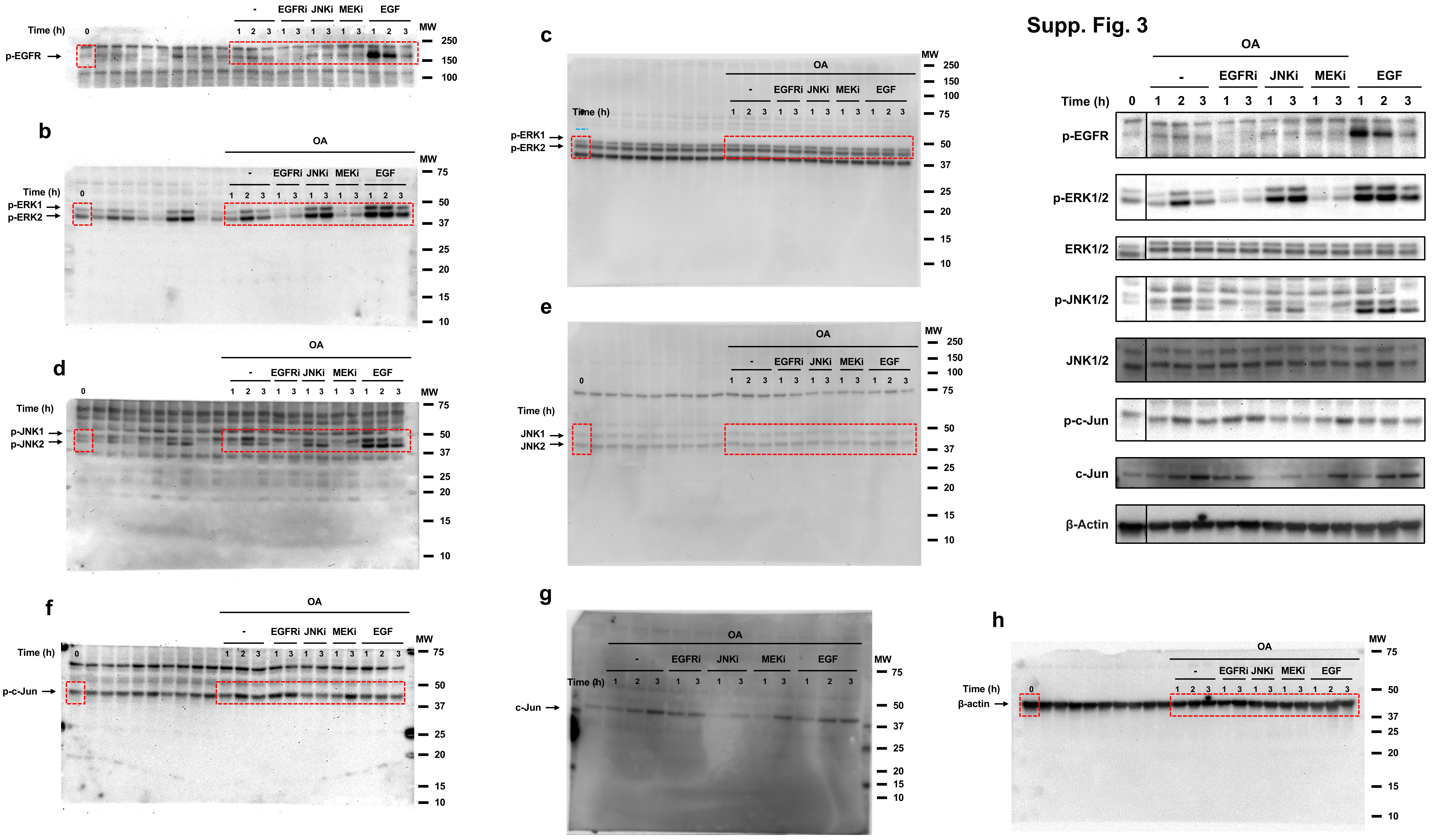

Supplemental Figure 11. Full-length blots corresponding to crops showed in Supplemental Figure 3. (a) Tyr 1068 Phosphorylated-EGFR. (b) Thr 202/Tyr 204 Phosphorylated ERK. (c) ERK1/2. (d) Thr 183/Tyr 185 Phosphorylated JNK. (e) JNK1/2. ((f) Ser 63 Phosphorylated c-Jun. (g) c-Jun. (h) Beta-actin loading. Dashed red rectangle indicates the portion of the blot that was used in the figure.
