# Supplementary material for: Oleanolic acid stimulation of cell migration involves a biphasic signaling mechanism
Source: Sci Rep. 2022 Sep 5;12:15065. doi: 10.1038/s41598-022-17553-w (PMC9445025; doi:10.1038/s41598-022-17553-w)
Supplement: Supplementary file 12 — Supplementary Figure 12. [file 41598_2022_17553_MOESM12_ESM.pdf]

Supp. Fig. 12

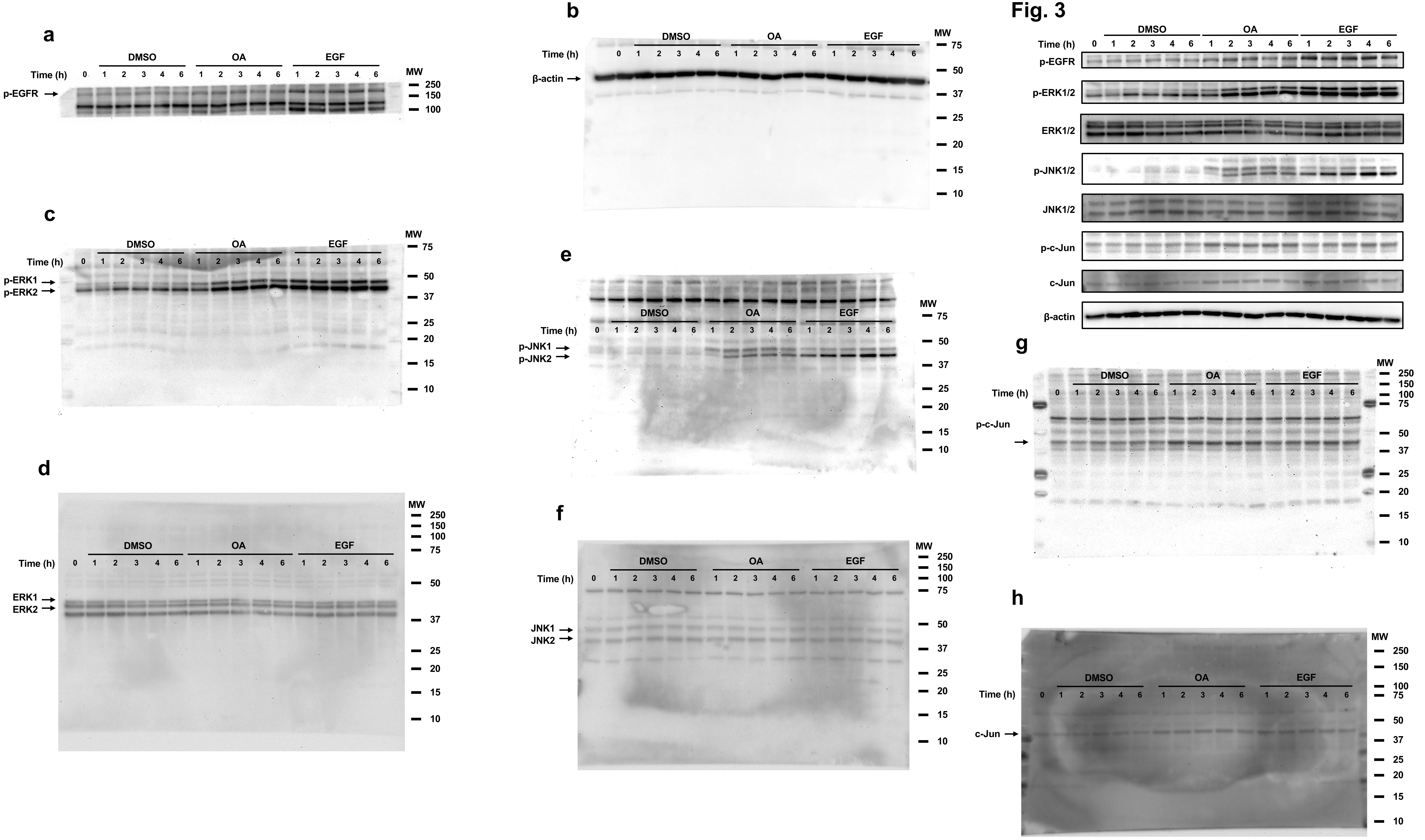

Supplemental Figure 12. Full-length blots corresponding to crops showed in Fig 3. (a) Tyr 1068 Phosphorylated-EGFR. (b) Beta-actin loading. (c) Thr 202/Tyr 204 Phosphorylated ERK. (d) ERK1/2. (e) Thr 183/Tyr 185 Phosphorylated JNK. (f) JNK1/2. (g) Ser 63 Phosphorylated c-Jun. (h) c-Jun.
