# Supplementary material for: Oleanolic acid stimulation of cell migration involves a biphasic signaling mechanism
Source: Sci Rep. 2022 Sep 5;12:15065. doi: 10.1038/s41598-022-17553-w (PMC9445025; doi:10.1038/s41598-022-17553-w)
Supplement: Supplementary file 13 — Supplementary Figure 13. [file 41598_2022_17553_MOESM13_ESM.pdf]

Supp. Fig. 13

a

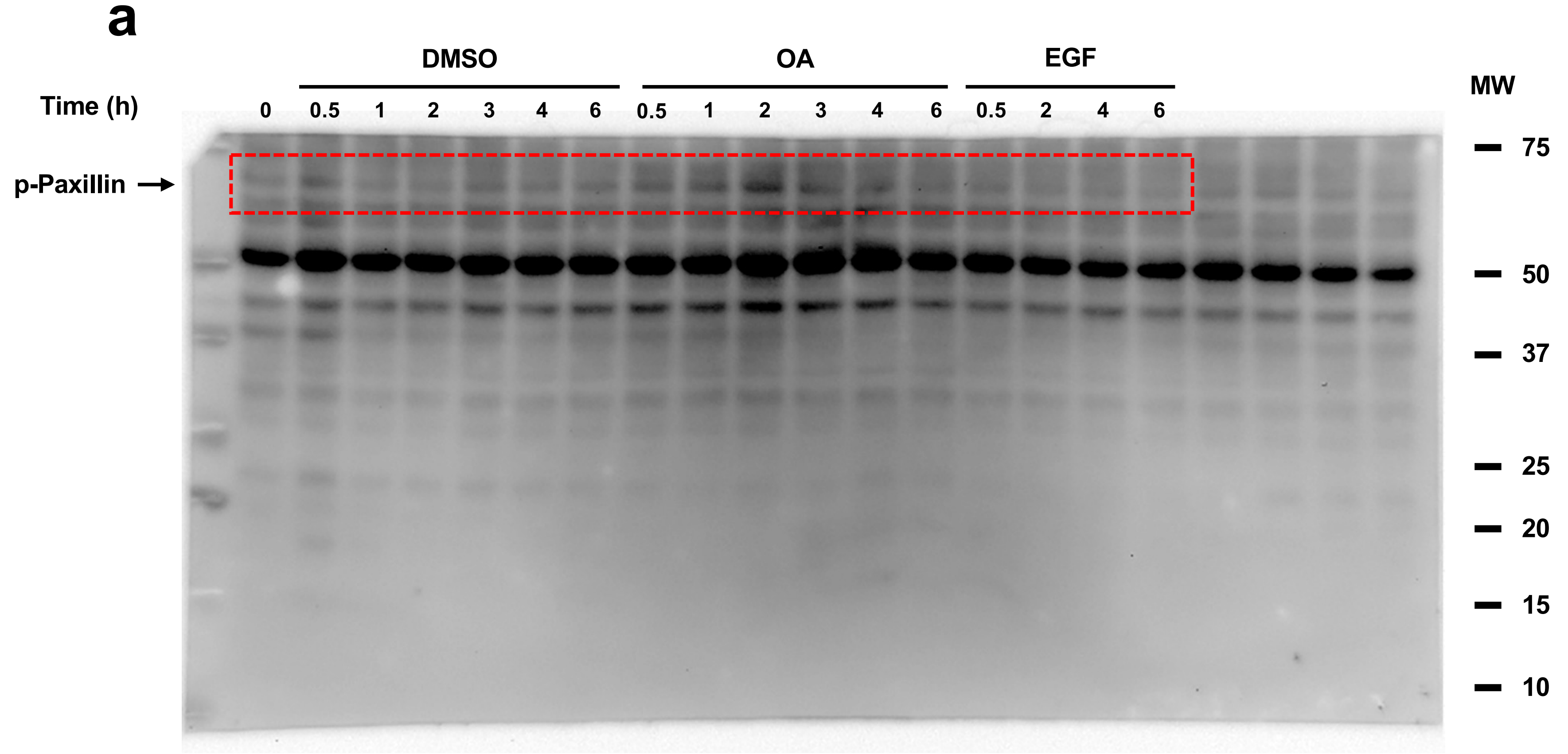

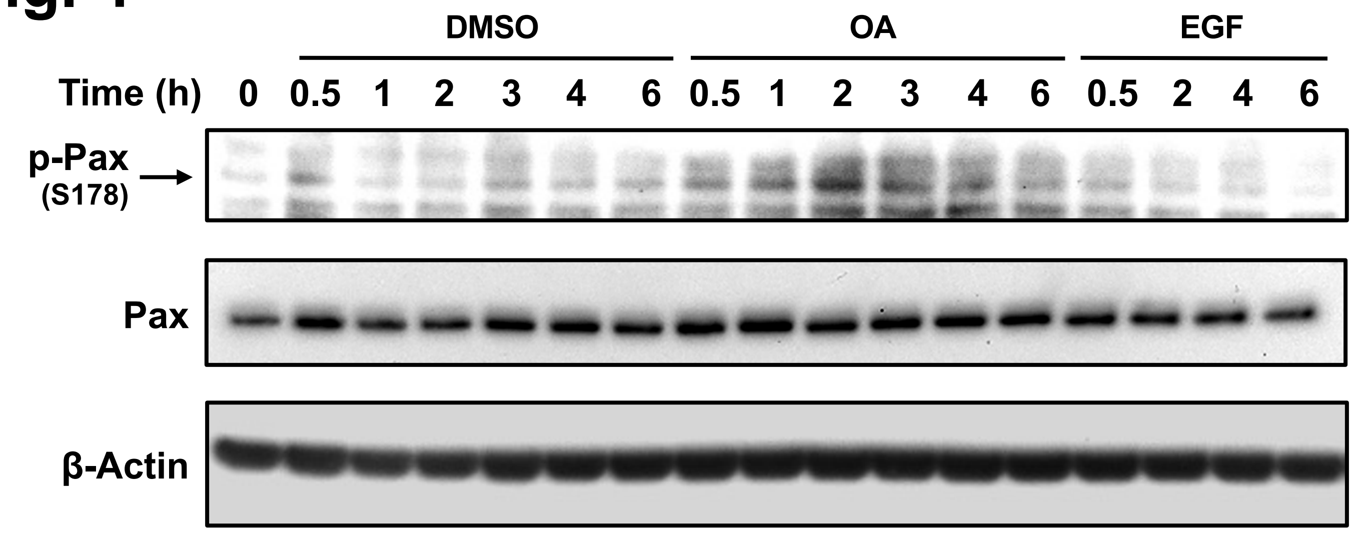

b

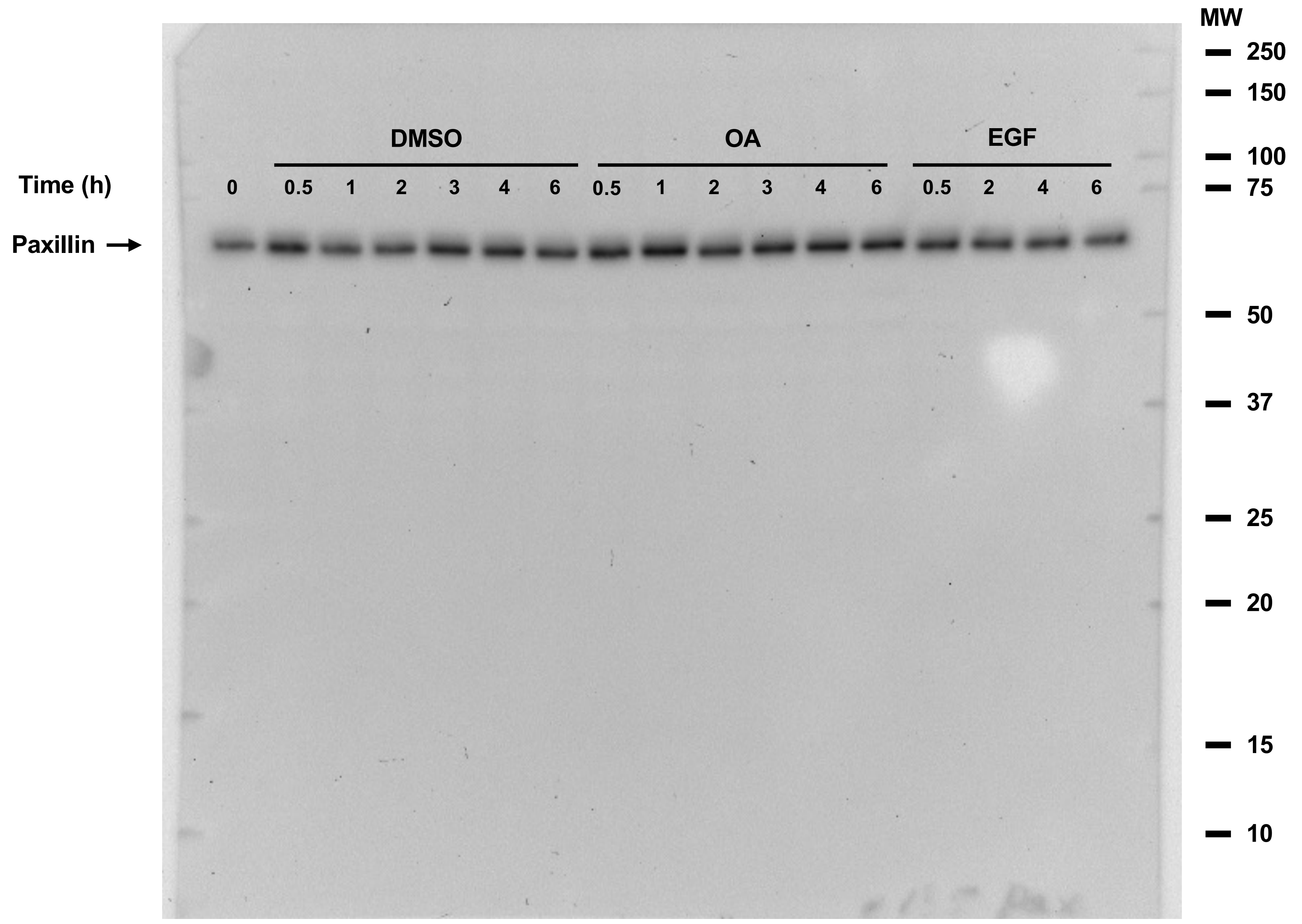

c

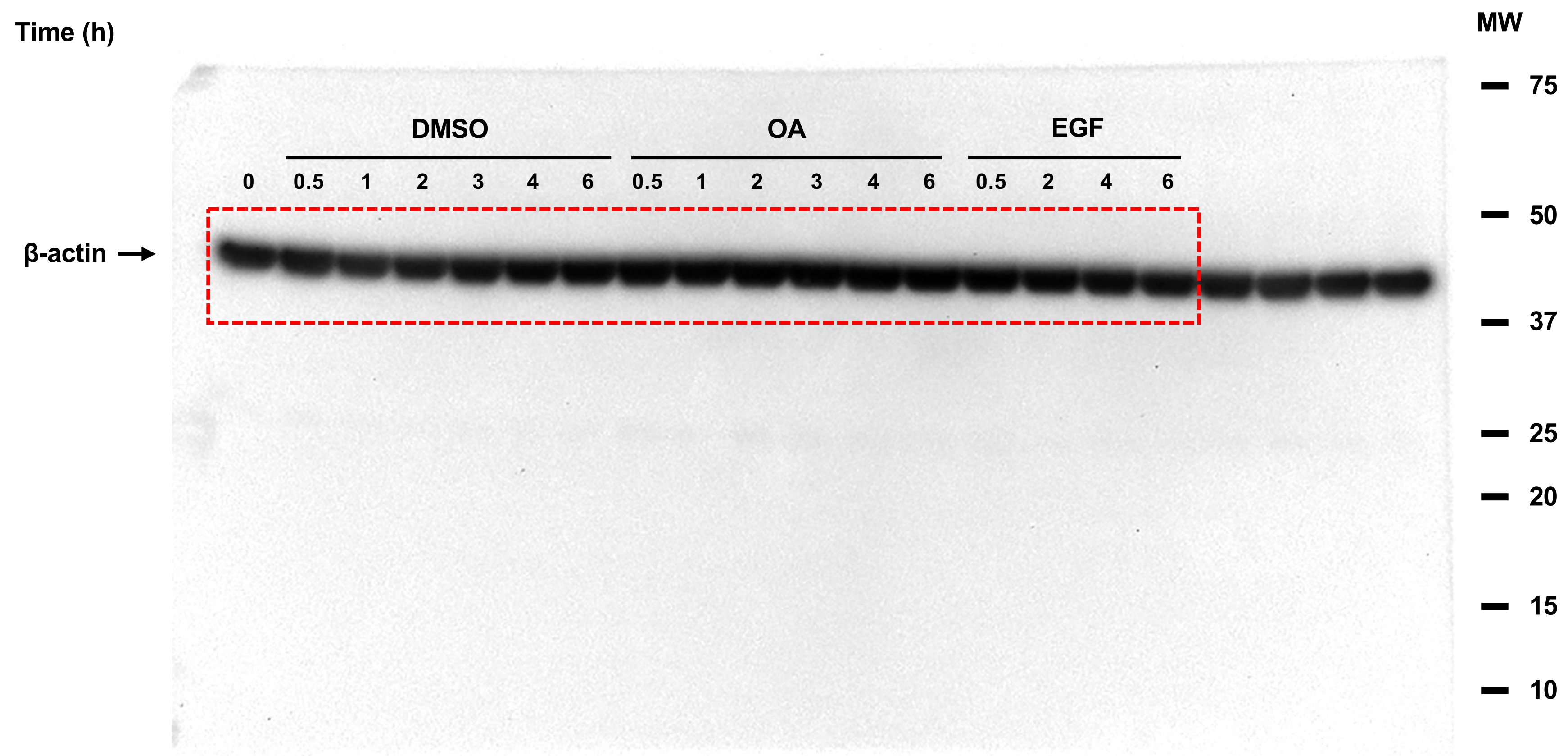

Supplemental Figure 13. Full-length blots corresponding to crops showed in Supplemental Figure 4. (a) Ser 170 Phosphorylated-Paxillin. (b) Paxillin. (c) Beta-actin loading. Dashed red rectangle indicates the portion of the blot that was used in the figure.
