# Supplementary material for: Oleanolic acid stimulation of cell migration involves a biphasic signaling mechanism
Source: Sci Rep. 2022 Sep 5;12:15065. doi: 10.1038/s41598-022-17553-w (PMC9445025; doi:10.1038/s41598-022-17553-w)
Supplement: Supplementary file 14 — Supplementary Figure 14. [file 41598_2022_17553_MOESM14_ESM.pdf]

Supp. Fig. 14

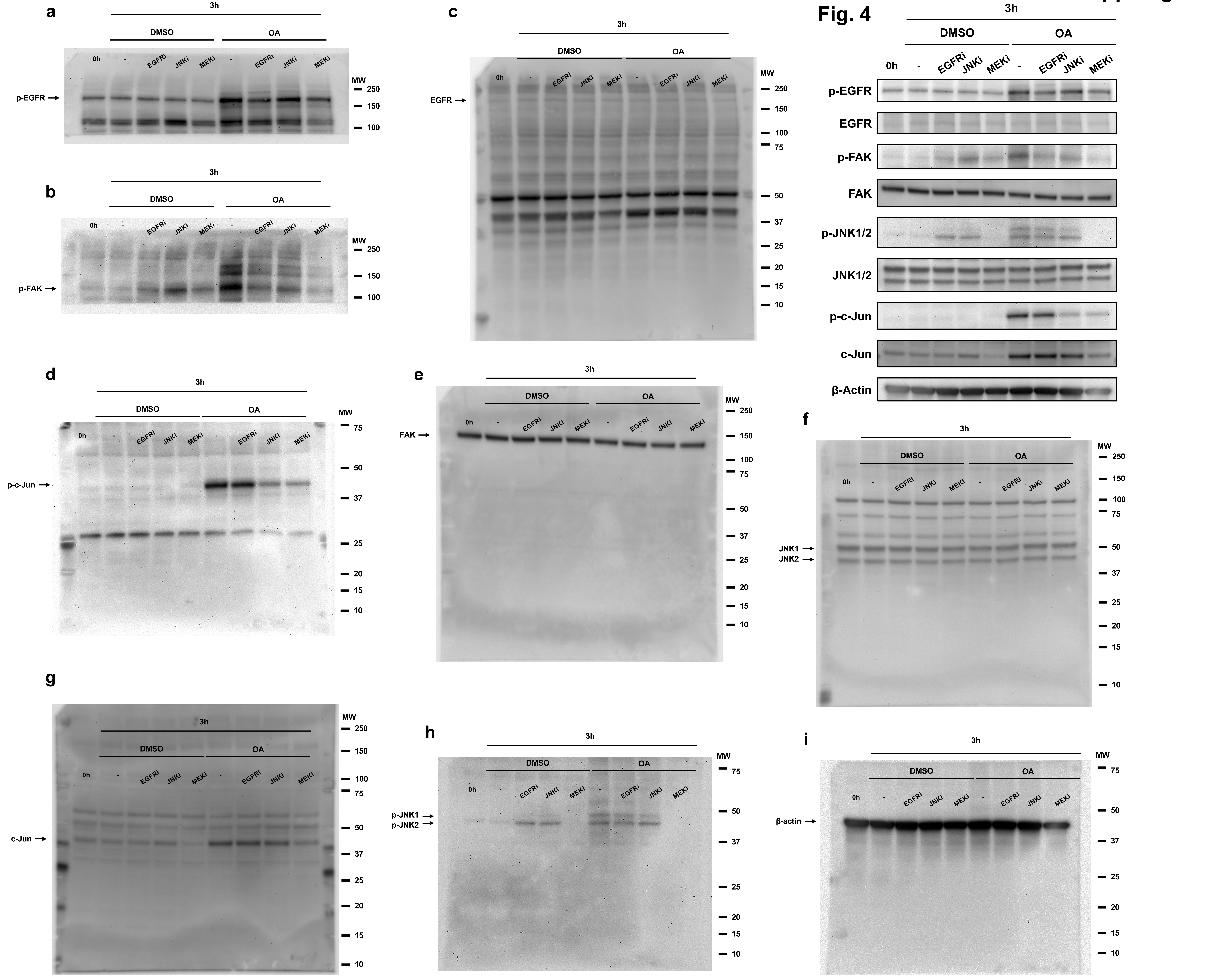

Supplemental Figure 14. Full-length blots corresponding to crops showed in Fig 4. (a) Tyr 1068 Phosphorylated-EGFR. (b) Tyr 925 Phosphorylated-FAK. (c) EGFR. (d) Ser 63 Phosphorylated c-Jun. (e) FAK. (f) JNK. (g) c-Jun. (h) Thr 183/Tyr 185 Phosphorylated JNK. (i) Beta-actin loading.
