# Supplementary material for: Oleanolic acid stimulation of cell migration involves a biphasic signaling mechanism
Source: Sci Rep. 2022 Sep 5;12:15065. doi: 10.1038/s41598-022-17553-w (PMC9445025; doi:10.1038/s41598-022-17553-w)
Supplement: Supplementary file 15 — Supplementary Figure 15. [file 41598_2022_17553_MOESM15_ESM.pdf]

Supp. Fig. 15

a

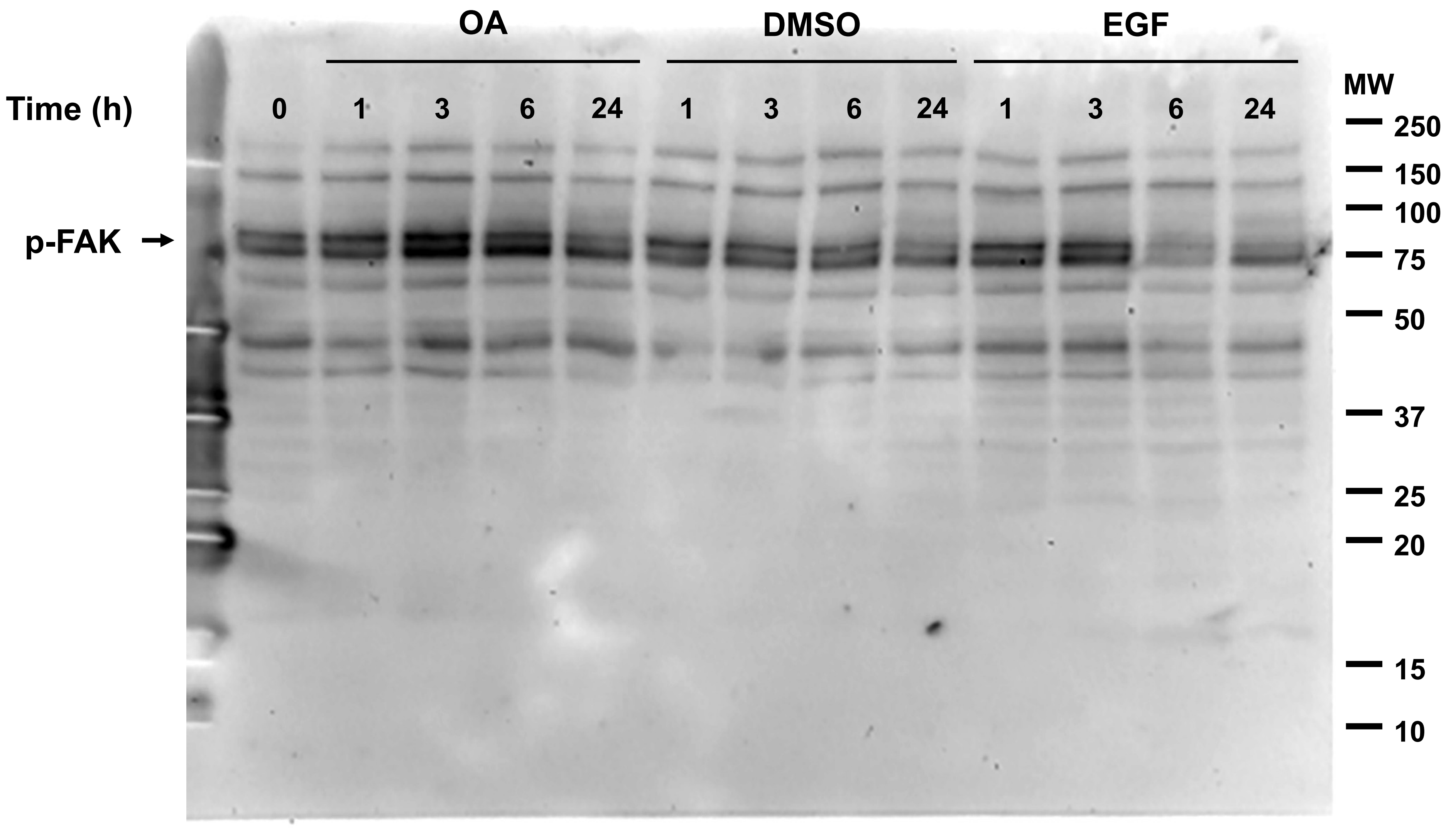

Fig. 7

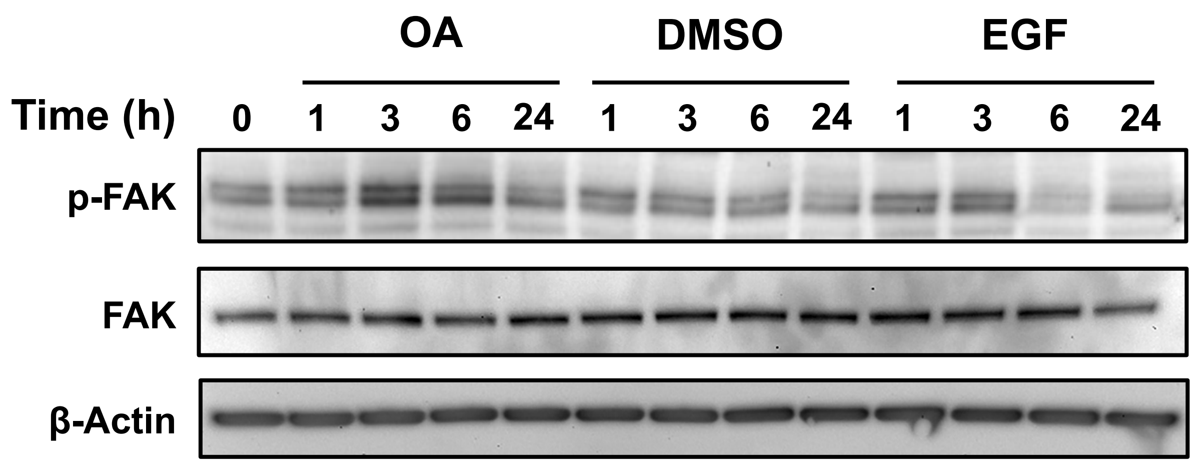

b

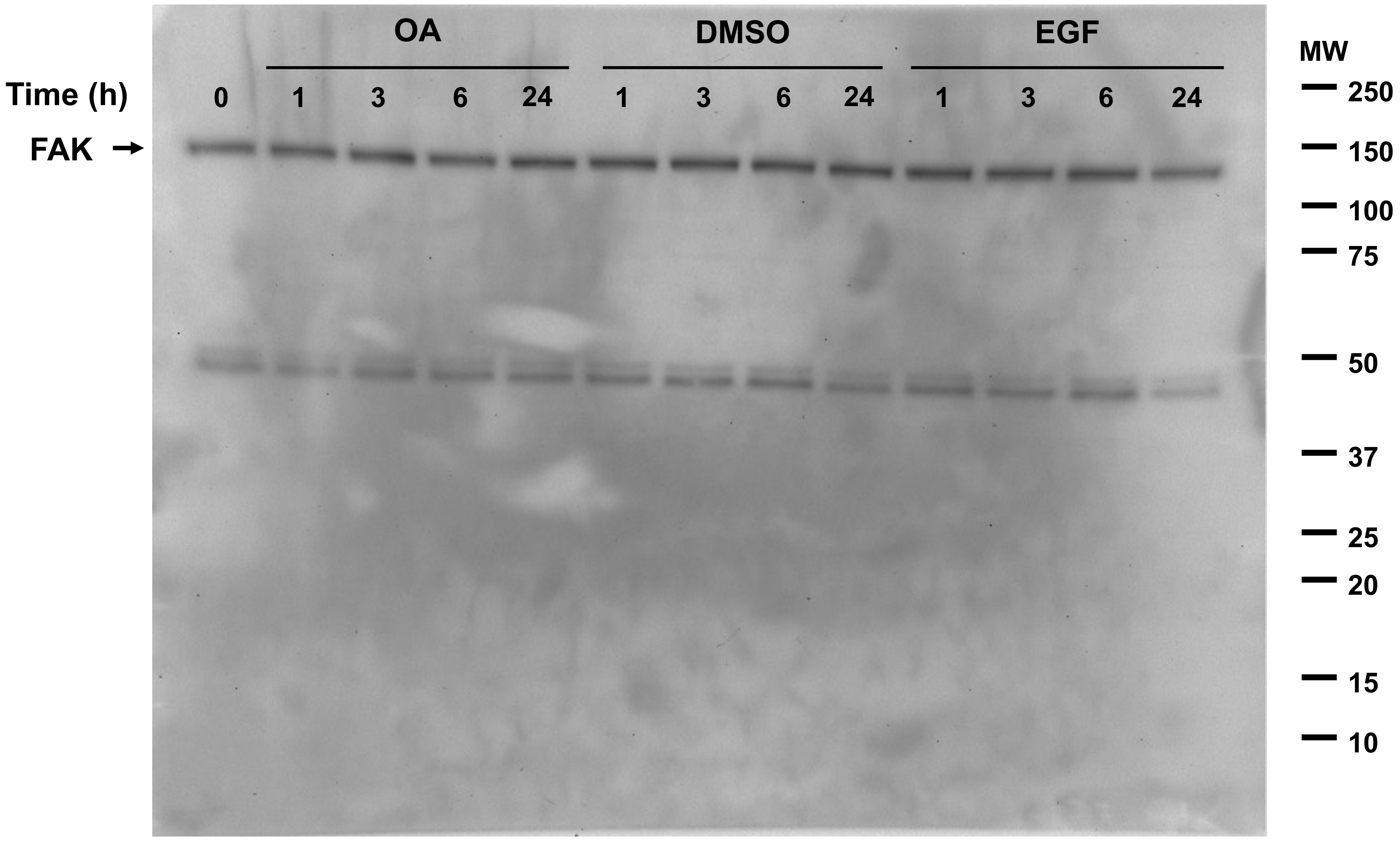

c

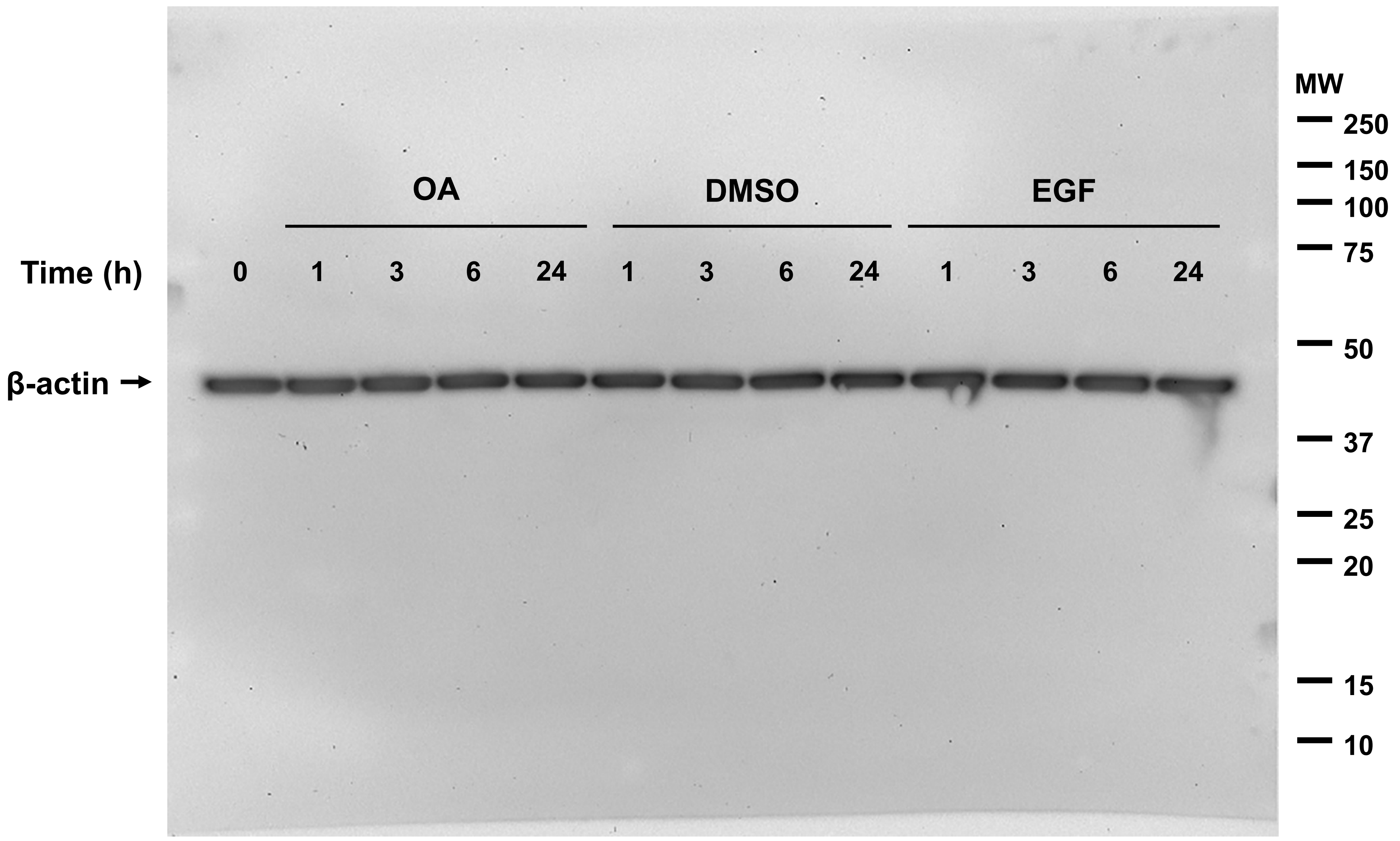

Supplemental Figure 15. Full-length blots corresponding to crops showed in Fig 7. (a) Tyr 925 Phosphorylated-FAK. (b) FAK. (c) Beta-actin loading.
